# An optimized spectral flow cytometry pipeline for longitudinal blood leukocyte immunophenotyping reveals stable, individually distinct immune signatures

**DOI:** 10.3389/fimmu.2026.1959895

**Published:** 2026-10-05

**Authors:** Katharine Gosling, Jason Price, David A. Simon Davis, Farhan M. Syed, Benjamin J. C. Quah

**Affiliations:** 1 Irradiation Immunity Interaction Lab, Division of Genome Sciences and Cancer, John Curtin School of Medical Research, Australian National University, Canberra, ACT, Australia; 2 Radiation Oncology Department, Canberra Hospital, Canberra Health Services, Canberra, ACT, Australia

**Keywords:** spectral flow cytometry, immunophenotyping, longitudinal monitoring, leukocyte composition, quality control, normalization, dimensionality reduction, biomarker discovery

## Abstract

**Introduction:**

Peripheral blood leukocyte immunophenotyping offers a minimally invasive insight into an individual’s immune system and is an attractive biomarker for detecting and monitoring disease. Advances in high-parameter spectral flow cytometry now resolve leukocyte subsets in unprecedented detail, but this depth adds complexity to sample preparation, acquisition and analysis, introducing technical variability that can obscure the biological signatures required for longitudinal monitoring.

**Methods:**

We built and validated an optimized, high-parameter spectral flow cytometry pipeline for repeated blood leukocyte profiling and asked whether the resulting signatures are stable and distinct enough to identify individuals over time.

**Results:**

Using rainbow beads and monoclonal-antibody spectra, we confirmed that instrument performance and spectral unmixing remained stable across more than two years, and that this dual measure localized faults to either the instrument or a reagent. Against a stabilized biological-invariant control, gating built on the dimensionality-reduction method PaCMAP was substantially more reproducible between operators than traditional two-dimensional gating and produced cleaner populations. Chemical fixation and one-week antibody pre-storage preserved leukocyte composition, whereas delayed processing and, more severely, cryopreservation altered it, the latter through near-complete granulocyte loss. Of the count-normalization schemes tested, normalization to the percentage of CD45^+^ leukocytes best preserved individual signatures over time, whereas counting-bead normalization performed worst. Applying the pipeline to ten donors sampled repeatedly across a year, we found that leukocyte signatures were dominated by stable, individually distinct differences between people — so distinct that a held-out visit could be assigned back to its donor, both by a trained random forest and by simple proximity to that donor’s own mean.

**Conclusion:**

Together these results define a technically sound, operator-reproducible and handling-robust pipeline whose normalized signatures capture a temporally stable individual immune fingerprint, providing a practical foundation for blood leukocyte profiling as a longitudinal biomarker of immune state in cancer and other diseases.

## Introduction

Peripheral blood is a readily accessible and minimally invasive source of information about an individual’s immune system, and the composition of circulating leukocytes carries signatures that reflect health, disease and treatment response (1–3). Since blood can be sampled repeatedly, it is particularly attractive for longitudinal monitoring and for the discovery of cellular biomarkers of cancer and other diseases, where serial tumor or tissue biopsy is impractical (2, 4). High-parameter spectral flow cytometry can resolve dozens of leukocyte populations in a single sample: by reading each probe’s full emission spectrum rather than one channel per fluorophore, it separates dyes with near-identical emission peaks but distinct overall spectra, enabling far larger marker panels (5, 6). This depth of phenotyping makes high-parameter spectral flow cytometry of blood cells an increasingly powerful technique for immune profiling.

Fulfilling this potential for longitudinal studies depends on the ability to detect changes in a leukocyte signature that reflect true biology rather than technical variation, a challenge that grows as assays become more complex (5). There are several sources of technical variability that could confound true biological leukocyte signature changes. Instrument performance and spectral-unmixing references can drift over time, degrading the consistency of high-parameter measurements (5). Population gating, the step that converts marker measurements into cell-population frequencies, is among the most subjective steps in the workflow, and manual two-dimensional (2D) gating introduces operator-dependent variability (7). Pre-analytical sample handling, such as delays before processing, cryopreservation and chemical fixation, can also alter the apparent leukocyte composition (8–11). Finally, raw cell counts often require a normalization strategy to be comparable across assays; counting-bead, volumetric and reference-population approaches are all used, but do not always agree (12).

Several protocol refinements offer pathways to identify and mitigate many of these problems. Instrument performance can be tracked by acquiring intensity-stable reference beads at each session and monitoring their signal across all detectors, while probe performance can be tracked by monitoring the emission spectrum of each fluorescent probe used in the assay. Together these provide ongoing quality control of instrument and probe variability (13). Subjective gating steps may be mitigated by unsupervised dimensionality-reduction algorithms such as Uniform Manifold Approximation and Projection (UMAP) (14) and Pairwise Controlled Manifold Approximation Projection (PaCMAP) (15): they condense high-dimensional marker space into two dimensions in which populations appear as discrete clusters for definitive gating. However, these algorithms are sensitive to stochastic initialization, raising the question of whether their cluster assignments are reproducible across independent experiments (15, 16). Pre-analytical variation from handling can be constrained either by analyzing longitudinally collected samples together in a single batch, which requires the cells to survive banking without compositional distortion, or by processing cells fresh at each timepoint while stabilizing other variables such as pre-prepared antibody cocktails (8–11, 17). Finally, between-run and between-batch scaling differences can in principle be corrected by count-normalization schemes anchored to counting beads, acquired volume, or a reference population such as the percentage of CD45^+^ leukocytes or a stabilized internal standard (12).

An individual’s immune system is shaped by both heritable factors and a lifetime of environmental exposure, and population-scale studies suggest this produces immune-cell compositions that are comparatively stable within a person yet markedly variable between people (1, 3, 18, 19). Whether such individuality persists in the routinely measurable blood leukocyte compartment, and on timescales relevant to clinical monitoring, remains to be established; establishing it requires first separating individuality from technical variation. By controlling these technical sources of variation, we aim to recover genuine biological change and to ask whether an individual’s leukocyte signature is stable across a year. If such an individual “immunological fingerprint” exists in routinely measurable blood leukocytes, it would both motivate and provide a stringent internal validation for longitudinal immunophenotyping as a biomarker for diseases such as cancer using spectral flow cytometry.

We therefore set out to build and validate an optimized pipeline for longitudinal blood leukocyte immunophenotyping by high-parameter spectral flow cytometry. We (i) established a dual-measure instrument and unmixing quality-control step that both verifies stability and localizes faults to instrument or reagent; (ii) compared PaCMAP-based and traditional 2D gating for operator reproducibility and gate cleanliness using a biological-invariant control, and defined a gating strategy on that basis; (iii) defined sample-handling practices that preserve leukocyte composition; (iv) determined which count-normalization scheme best preserved longitudinal signal, first by its effect on compositional stability over time, then functionally, by whether it preserved random-forest re-identification of individual donors; and (v) asked whether the resulting signatures are dominated by stable, individually distinct inter-donor differences. Together these aimed to define a technically sound, operator-reproducible, handling-robust and signal-preserving spectral flow cytometry pipeline, enabling a temporally stable individual immune fingerprint as a potential biomarker for disease states such as cancer.

## Materials and methods

### Participants and ethics

For the longitudinal study, ten healthy adult donors were sampled across up to six visits at approximately two-monthly intervals; whole blood was collected by venipuncture into K2E (EDTA) vacutainers (367525, BD Biosciences). The blood sample-handling optimization used blood collected from a subset of the ten donors prior to the commencement of the longitudinal study. This study used procedures approved by the Australian National University Human Research Ethics Committee (ANU HREC protocol 2022/541); all participants gave written informed consent.

### Blood processing and flow cytometry acquisition

Human blood samples were red-blood-cell–depleted by adding 5mL of 37°C pre-warmed 1X BD Pharm Lyse lysing buffer (555899, BD Biosciences) to 1mL blood and incubating on an orbiting platform (500rpm) for 20 minutes at room temperature. Lysis was stopped by addition of 6mL of cold PBS (20012050, Gibco), then the samples were centrifuged for 10 minutes at 300 x g. Supernatant was removed and the cell pellets resuspended in PBS to 1mL. 100μL of each sample was transferred to a 96 well plate along with 100μL of PBS containing approximately 5000 counting beads (Coulter Flow Count, 7547053, Beckman Coulter). The plate was centrifuged (5 minutes, 300 x g, 4°C) and supernatant discarded. Blood leukocytes were incubated for 10 minutes with Fc receptor blocking solution (Human TruStain FcX, 422302, BioLegend) and then labeled with a 44-marker fluorescent monoclonal-antibody (mAb) panel (**Supplementary Table S1**) overnight at 4°C on an orbiting platform; this timing was adopted based on recently reported extensive optimization (20). Cells were then incubated for 10 minutes at room temperature with Zombie NIR fixable viability stain (423105, BioLegend), then washed 3 times with PBS/5mM EDTA (03690, Sigma)/1% BSA (A7030, Sigma) (centrifuging 5 minutes, 300 x g, 4°C). Cells were resuspended in a total volume of 150μL and acquired on a Cytek Northern Lights spectral analyser (Cytek Biosciences); 100μL of each cell suspension was collected at Medium speed using the same saved user settings each time. For the blood sample-handling optimization experiments, cells were labeled with a reduced mAb panel and were mainly collected on an LSRFortessa X-20 flow cytometer (BD Biosciences). Because the number of informative markers exceeded the number of spectrally-distinct fluorochromes resolvable by unmixing, twelve fluorochromes were each assigned to two or three antibodies whose target antigens are read out within different leukocyte lineages (**Supplementary Table S1**), a shared-fluorochrome strategy used previously to record more markers than available fluorochromes (21), including by our group (4). As each fluorochrome contributes a single spectral signature to unmixing, these antibodies were resolved not spectrally but by lineage-restricted gating, the signal being interpreted as the marker relevant to the population in which it was gated. Assignments placed co-assigned antigens in non-overlapping branches of the gating hierarchy and were validated using single-stain spectral reference controls (using SpectraComp mAb binding beads; SSB-05-B, SlingshotBio, and ViaComp vitality dye binding beads; SSB-07-A, SlingshotBio) and lineage-gated fully-stained samples.

### CD-CHEX Plus controls

CD-CHEX Plus stabilized human blood (213323, lots 32680071, 41550071, 50690071 and 53210071, Streck) was used as a biological-invariant control. 100-200μL from each CD-CHEX tube was sampled as per the manufacturer’s instructions and red cell lysis proceeded as for the human blood samples, with 5 volumes of lysis buffer to 1 volume of cells followed by 6 volumes of chilled PBS to stop lysis. Staining, washing and acquisition was as for the human blood samples but without the viability marker staining.

### Instrument/unmixing quality control

To monitor (spectral) instrument and unmixing stability across the study, two quality-control (QC) measures were tracked on each of 38 acquisition dates spanning approximately 32 months. First, bead-captured (SpectraComp beads, SSB-05-B, SlingshotBio) single-stain mAb reference spectra were recorded and the spectral signature of each fluorochrome on each date was compared with the study reference using cosine similarity [typical of Cytek similarity indexes (22)], expressed as a dissimilarity (1 − cosine similarity). Second, 8 peak Sphero Rainbow Calibration Particles (559123, BD Biosciences) were acquired and the per-detector coefficient of variation (%CV) of each bead peak (RB-1 to RB-8) was compared with the reference in the same way. Each RB peak was gated according to the gating strategy depicted in **Supplementary Figure S1A**. Acquisition dates were flagged for review where spectral or bead-CV dissimilarity departed markedly from a predetermined historical baseline. Cosine similarity measures how closely two profiles match in *shape*: the cosine of the angle between them when each is treated as a vector across detectors, independent of overall magnitude; it ranges from 1 (identical shape) to 0 (orthogonal). Dissimilarity was expressed as 1 − cosine similarity, so 0 indicates a profile identical in shape to the reference and 1 a maximally divergent one and is thus suited to detecting spectral drift.

### Population gating, PaCMAP and gating-consistency assessment

To compare the consistency of PaCMAP-based and traditional 2D hierarchical biaxial gating for leukocyte subset identification, CD-CHEX Plus stabilized human blood was used. CD-CHEX Plus stabilized blood contains fixed leukocytes whose composition cannot change over time, so any variability in gating outcomes reflects the gating methodology rather than biological change (Streck). Three expert operators each independently gated eight major leukocyte populations (CD4^+^ T cells, CD8^+^/γδ T cells, NK cells, B cells, neutrophils, monocytes/macrophages/dendritic cells, eosinophils and basophils) using (i) traditional hierarchical 2D biaxial gating and (ii) PaCMAP (Pairwise Controlled Manifold Approximation Projection) dimensionality-reduction-based gating. All gating and flow cytometry analysis was performed in R using the CytoExploreR package (23) using the gating strategies depicted in **Supplementary Figures S1B, C**. UMAP-defined reference gates, drawn by a single expert, served as the population reference embedding against which gating purity was assessed (**Supplementary Figure S1E**); UMAP is treated here as the reference embedding, not as absolute ground truth. No random seed was set at any stage of the PaCMAP or UMAP implementation, so each dimensional reduction was randomly initialized. However, each acquisition timepoint used a single randomly initialized PaCMAP embedding, common to all three operators, so that the inter-operator comparison with 2D biaxial gating — where every operator necessarily viewed identical plots — reflected gating judgement alone.

For each gated population from each method, the percentage of cells falling within the corresponding UMAP reference gate was computed using `cyto_stats_compute()` in CytoExploreR, yielding a correct-assignment percentage (diagonal) and a contamination matrix (off-diagonal; i.e. incorrect-assignment) for each operator × method × timepoint combination (as depicted in **Supplementary Figures S1F, G**). Temporal stability was quantified as the standard deviation (SD) of correct-assignment percentages across timepoints for each operator × method × population combination, restricted to donor–timepoint combinations with ≥ 2 timepoints. Inter-rater agreement was assessed using the intraclass correlation coefficient (ICC; two-way mixed effects, absolute agreement, single measures, ICC(A,1)) computed with the irr package (24), per donor × method × population, with timepoints as subjects and operators as raters; groups with < 2 timepoints or < 3 raters were excluded, as were degenerate groups in which raters agreed perfectly (ICC undefined). Contamination was defined as the percentage of cells from a gated population landing in a non-corresponding UMAP reference gate; the proportion of zero-contamination events (gate cleanliness) and the mean contamination level were compared between methods. Differences between methods were tested with Wilcoxon rank-sum tests and Benjamini–Hochberg (BH) false-discovery-rate correction across the six metrics, with the per-donor × population combination as the unit of observation.

Within each of the eight main leukocyte populations of the human donor blood (CD4^+^ T cells, CD8^+^/γδ T cells, NK cells, B cells, neutrophils, monocytes/macrophages/dendritic cells, eosinophils and basophils), subpopulations were further delineated using manual gates (depicted in the total finalized leukocyte gating strategy in **Supplementary Figure S1H**) to give 36 subpopulations (defined in **Supplementary Data S1**).

### Blood sample-handling conditions

Whole blood from healthy donors was used to assess the impact of four pre-analytical variables on leukocyte immunophenotyping: (i) delayed processing following storage at 4°C (1, 2 or 3 days); (ii) cryopreservation in liquid nitrogen using either CryoStor CS10 (C2784, Sigma) or 20% dimethyl-sulfoxide (DMSO, 2225-500, Ajax FineChem)-80% foetal bovine serum (F8192, Sigma), assessed at Day 15 post-freezing; (iii) chemical fixation using the Cytofix reagent from BD Cytofix/Cytoperm™ Plus kit (555028, BD Biosciences), analyzed at Day 1 and Day 5 post-collection and compared with unfixed Day 1 samples; and (iv) antibody cocktails stored at 4°C for one week compared with freshly prepared antibodies in BD Horizon™ Brilliant Stain Buffer Plus (568264, BD Biosciences). Each condition used n = 3 replicates and was assessed for blood leukocyte composition using mAb labelling and flow cytometry similar to the techniques described above, but with a truncated mAb panel. These n = 3 replicates were technical replicates prepared from split samples, so each condition estimates the effect of a handling variable on a given blood sample; between-donor variation in the magnitude of that effect was not sampled. Five leukocyte lineages (T cells, monocytes/macrophages/dendritic cells, B cells, eosinophils and neutrophils) were harmonized across samples gated to differing depths. T-cell counts were taken directly where available, or otherwise inferred, in order of preference, as live CD45^+^ cells minus non-T cells, or as the sum of the CD4^+^, CD8^+^ and γδ (or CD4^+^ and CD8^+^) T-cell subsets; monocyte/macrophage/dendritic-cell counts were taken from the combined gate where present and from the monocyte gate otherwise; B-cell, eosinophil and neutrophil counts were taken directly. All samples within an experiment were harmonized to the same set of subsets. Complete data-processing code is available in the accompanying repository (script 03_sample_handling_optimisation_and_two_tier_normalisation. R). Leukocyte subset composition was expressed as proportions of total leukocytes. The change in each group was computed as the difference in percentage points from the mean reference-samples value for each population. Compositional differences between conditions were statistically assessed by permutational multivariate analysis of variance [PERMANOVA; vegan, `adonis2`, 999 permutations (25)], using Bray–Curtis dissimilarity for the five-population subset analysis. Given n = 3 per group, PERMANOVA was preferred over parametric alternatives as it makes no distributional assumptions and is robust at small sample sizes.

### Count normalization

Leukocyte quantities were expressed under a panel of normalization schemes: raw gated event counts (Raw) and two successive normalization tiers (Tier 1 and Tier 2), each computed on one of three bases: the percentage of CD45^+^ leukocytes (%CD45), a spiked counting-bead ratio (bead; gated as defined in **Supplementary Figure S1H** panel 1 and 2), or the acquired sample volume (vol). Tier 1 rescaled the raw count within each sample: %CD45 = count/total CD45^+^ × 100; bead = count × (beads added/beads counted) × 10 (to express per mL); vol = count × (well volume/collection volume) × 10 (to make to/mL). Tier 2 additionally applied a cross-batch CD-CHEX correction (corr) factor to place counts on a common, drift-corrected scale (Tier 2 = Tier 1 × corr(date, lineage)). The correction factor was built as a product chain, Tier 2 = FC · R(L) · G(p), where FC = sample/C_L(p,e) is the fold change versus the same-day CD-CHEX value, removing the run effect (C_L(p,e) being the geometric mean of CD-CHEX vials for lot L and population p in experiment e); R(L) = ∏ f is the cumulative re-anchor factor, the product of step bridge factors f = C_new/C_old chained back to the anchor lot; and G(p) is the global anchor, the geometric mean of the anchor lot’s values for population p across its runs (equivalently, corr(date, lineage) = R(L) · G(p)/C_L(p,e)). Subpopulation counts were closed within their lineage (proportion = subpopulation/Σ subpopulations within the lineage) and rescaled to the anchored lineage count; geometric means were used at each averaging step. For further clarity, this 2-Tier scheme is depicted graphically in **Supplementary Figure S2**.

### Longitudinal stability of normalization schemes

The effect of each normalization scheme on longitudinal stability was assessed in two independent datasets: held-out CD-CHEX lots acquired across the study as an independent stability control, and repeated fresh sampling of the same ten donors. Compositional drift was quantified as the Bray–Curtis dissimilarity of every timepoint to the first (the first acquisition for each CD-CHEX lot) and each donor’s first visit. This was assessed for the CD-CHEX lineage populations and, in the donor dataset, for both the leukocyte lineages and the finer subpopulations (lower and flatter trajectories indicate greater stability). Bray–Curtis dissimilarity measures the compositional difference between two samples as the sum of absolute differences in population abundance divided by their combined total abundance; it ranges from 0 (identical composition) to 1 (no shared populations) and is thus suited to detecting shifts in the relative sizes of leukocyte populations.

Each Tier scheme was also compared against raw counts within Bayesian Beta regressions [brms (26)], fitted separately for the CD-CHEX-lineage, donor-lineage and donor-subpopulation datasets. Bray–Curtis dissimilarity (clamped to the open interval [1×10^−4^, 1 − 1×10^−4^]) was modelled as dissimilarity ~ scheme + (1 | population group) with a Beta likelihood, weakly-informative priors (Normal(0, 1) on the intercept and coefficients; Exponential(1) on the group-level standard deviation), and fitted with four chains of 6,000 iterations (2,000 warmup; 16,000 posterior draws; adapt_delta = 0.99; seed 42). For each posterior draw, the difference in modelled dissimilarity between a scheme and raw counts was computed on the response scale (ΔBray–Curtis; negative values indicating greater stability than raw counts), giving a posterior distribution of that difference. We report the posterior median, 80% and 95% credible intervals (CI), and the posterior probability that a scheme was more stable than raw (P; the proportion of draws with ΔBray–Curtis < 0). A scheme was termed more or less stable than raw only where its 95% CI excluded zero; schemes whose CI crossed zero were treated as equivalent to raw counts. Convergence (Rˆ, effective sample size, divergent transitions) and fit (Bayesian R²) were checked for all models and are reported in **Supplementary Data S2**.

### Random-forest donor re-identification

To test which normalization scheme best preserved donor-discriminating signal, random forests [ranger (27, 28)] were trained to predict donor identity from the leukocyte signature of a held-out visit (blood sample), using leave-one-visit-out cross-validation. Ten donors were each sampled across six visits at approximately two-monthly intervals; chance accuracy was therefore 1/10 = 0.10. Models were trained separately for each normalization scheme and for three feature sets: leukocyte lineages alone (8 features), subpopulations alone (36 features), and the two combined (44 features), and repeated across 25 random seeds per condition, with fold accuracies averaged over seeds. Each forest comprised 500 trees grown in probability mode with the Gini split rule and a minimum node size of 10; the realized mtry was 2 for the lineage feature set and 6 for the subpopulation and combined sets, with a single thread set for reproducibility. The top-3 accuracy counted the true donor among the three highest-probability classes; folds with fewer than four test rows or eight training rows were skipped. Performance was summarized as classification accuracy (primary), top-3 accuracy and macro-F1, each reported as the mean over seeds × folds with the 2.5–97.5 percentile spread (**Supplementary Table S2**).

### Nearest-centroid donor re-identification

To test the longitudinal stability of the different normalization scheme readouts directly, rather than through a learned non-linear decision boundary, donor identity was also predicted by nearest-centroid assignment under the same leave-one-visit-out scheme, normalization schemes and feature sets used for the random forest classifier defined above. Each feature (f) is one leukocyte population expressed under the scheme being tested. Within each training fold, features with no variance were dropped and the remaining features (F) standardized as z-scores, z(f) = (x(f) − m(f))/s(f), where x(f) is the feature value and m(f) and s(f) are that feature’s mean and standard deviation across all training samples, pooling donors and visits; held-out samples were transformed with the same m(f) and s(f) and contributed to neither. Standardizing to z(f) units means each feature contributes in proportion to its variability across the cohort, rather than the distance being dominated by the most abundant populations. A centroid was then computed for every donor (d) by averaging the z(f) values across that donor’s n training visits (tv), giving one value per feature, c(f,d) = (1/n) Σ z(f,tv). The held-out sample (hv) was then assigned to the nearest of the ten donor centroids by Euclidean distance, D(hv,d) = √[Σ (z(f,hv) − c(f,d))²], the sum running over all F retained features (F = 44 at Tier 1%CD45 with the combined feature set). A fold was scored only where at least eight training samples and four held-out donors remained after incomplete records were removed, giving five folds for the subpopulation and combined feature sets and six for lineages alone. The classifier is deterministic, with no random seed, no hyperparameters and no tuning step. Both classifiers were scored on identical folds, so any difference between them reflects the decision rule rather than training variance. Performance was summarized as accuracy, and assignments at Tier 1%CD45 with the combined feature set are displayed as a matrix of true against assigned donor.

### Bayesian binomial contrast models

Pairwise differences in donor-re-identification accuracy between normalization schemes, and between feature sets, were estimated using Bayesian binomial models [brms (26)]. Seed-averaged fold accuracies were modelled as binomial outcomes (n_correct | trials(n_test) ~ predictor + held-out visit), with the held-out visit as a random intercept where four or more folds were available and as a fixed effect otherwise; the predictor was normalization scheme (versus raw) or feature set (versus lineages). Weakly-informative Normal(0, 1.5) priors were placed on the intercept and coefficients (and Exponential(1) on the visit random-effect standard deviation), and models were fitted with the same MCMC settings as the Beta models above (four chains of 6,000 iterations; 2,000 warmup; 16,000 posterior draws; adapt_delta = 0.99; seed 42). For each contrast we report the median accuracy difference with 80% and 95% CI and the posterior probability of the difference being in the stated direction, P(direction). A difference was termed credible only where its 95% CI excluded zero; contrasts with high P(direction) but a 95% CI crossing zero are described as not credible under this criterion. The same framework was used to compare the two classifiers. Because nearest-centroid and random-forest predictions were generated on identical folds, the fold-level counts from both were fitted in a single model, n_correct | trials(n_test) ~ classifier × normalization scheme + (1 | held-out visit), so that the classifier contrast is estimated within held-out visit and the interaction allows the difference between classifiers to vary by scheme. Posterior accuracies were taken on the response scale with the visit random effect marginalized out, and the reported contrast is the nearest-centroid minus random-forest difference at each scheme, summarized with the same median, 80% and 95% CI and P(direction) as above. Convergence and fit were checked for all models and are reported in **Supplementary Data S3**.

### Inter- versus intra-donor signature variance

Analyses used Tier 2%CD45 counts, the combined lineage-and-subpopulation feature set, and Bray–Curtis dissimilarity (defined above). Donor mean compositions were displayed as a heatmap clustered by population and donor [Ward.D2 on Bray–Curtis distances; ComplexHeatmap (29)]. Two dissimilarity readouts were computed across visits: intra-donor, each visit versus that donor’s own first visit; and inter-donor, each donor’s mean dissimilarity to all other donors at the same visit. Compositional variance was partitioned between donor and timepoint by PERMANOVA (vegan adonis2, marginal terms) with permutations restricted within donor (how(blocks = donor), 9,999 permutations); this validly tests the timepoint term, so the donor term is reported as an effect size (R²) rather than a p-value. Homogeneity of multivariate dispersions was confirmed for both groupings (betadisper/permutest), and intra- versus inter-donor dispersions were compared as the mean distance to the group centroid by a one-sided permutation test (H1: inter > intra). Bray–Curtis distances were ordinated by PCoA to show intra-donor (per-donor hulls) and inter-donor (per-timepoint hulls) spread; leukocyte populations were fitted by envfit (999 permutations), with the eight most strongly associated (p ≤ 0.05) shown as vectors on both panels.

### Software

All analyses were performed in R 4.4.3 using CytoExploreR 2.0.12, irr 0.84.1 (24), tidyverse 2.0.0 (30), vegan 2.6-10 (25), ranger 0.17.0 (28), brms 2.22.0 (26), ComplexHeatmap 2.22.0 (29) and patchwork 1.3.2.9000 (31); cosine similarities for the spectral QC were computed with lsa 0.73.3 (32).

### Use of generative AI

Anthropic’s Claude (Claude Opus 4.8; Anthropic, 2025–2026), accessed through its chat, Claude Code and Cowork interfaces, was used to assist with drafting and editing manuscript prose and figure legends, structuring and reviewing analysis scripts, and formatting reference metadata. It was not used to generate, analyze, or interpret primary data, or to produce any figures or images directly. All AI-assisted output was reviewed, verified and edited by the authors, who confirm that all hypotheses, results, interpretations and conclusions are their own and take full responsibility for the accuracy and integrity of the work.

## Results

### Complementary QC measures confirm instrument and unmixing stability and distinguish instrument from reagent faults

The global aim of this study was to monitor the stability of the blood leukocyte profile of donors across a 12-month period using a spectral flow cytometry pipeline. Before any flow cytometry-generated signature can be trusted across a longitudinal study, the measuring instrument and the spectral-unmixing references must themselves be stable over time (5). We therefore tracked two independent quality-control (QC) measures across 38 acquisition dates spanning approximately 32 months: the spectral signature of each fluorochrome in our final monoclonal antibody (mAb) panel on capture beads (rather than experimental cells that may vary), and the per-detector coefficient of variation (%CV) of the 8 fluorescent peaks (RB-1 to RB-8) of rainbow calibration beads (RB), each compared with the study reference and expressed as a dissimilarity from this reference (1 − cosine similarity; 0 = identical to reference, 1 = maximally dissimilar) (**Figure 1A**). The two readouts isolate two potentially different failure modes: RB CV deviations reflect instrument stability, whereas mAb spectral signature deviations additionally report on reagent integrity, so their concordance or dissociation localizes anomalies to the instrument or to the mAbs.

**Figure 1:**
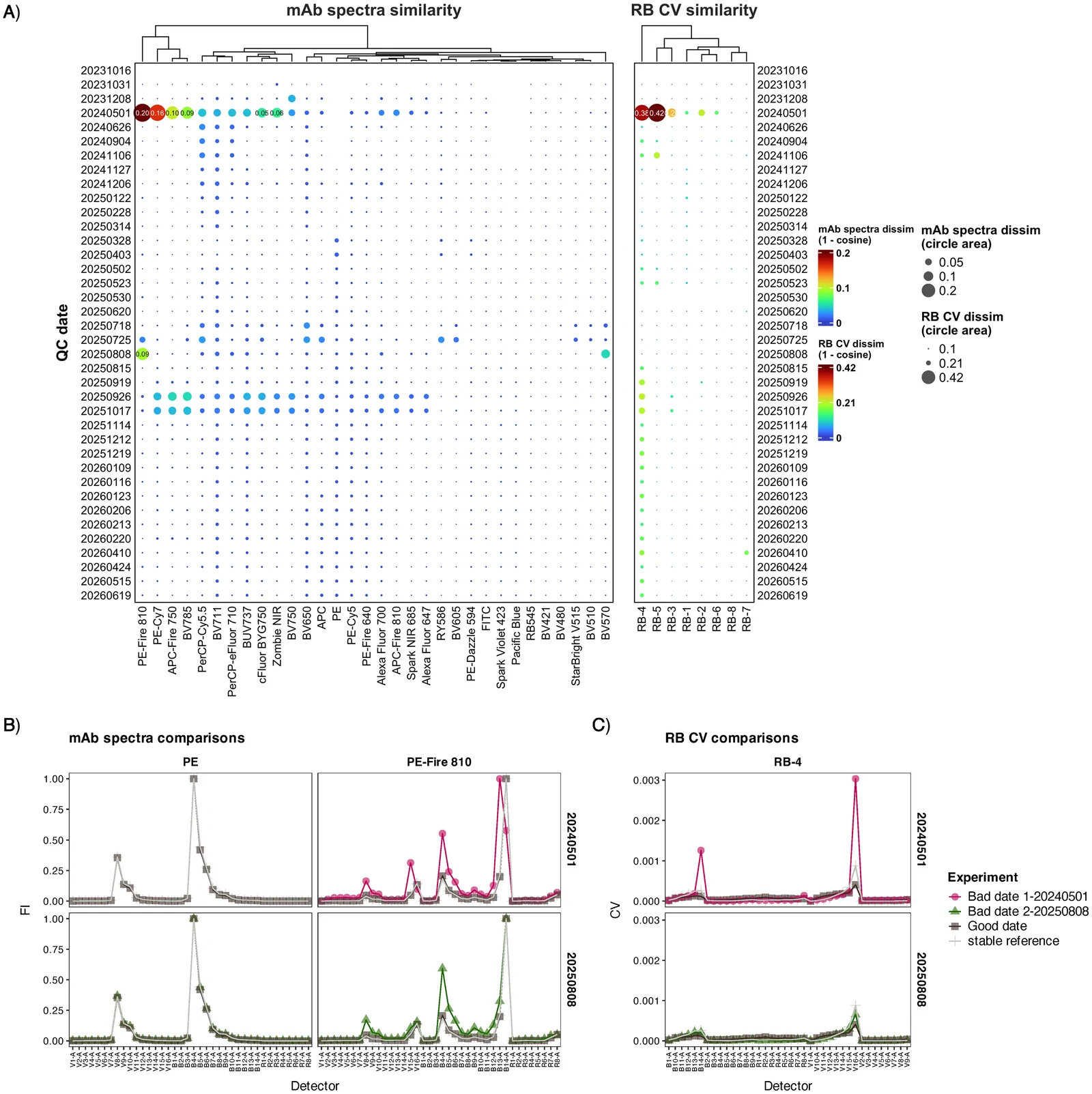
Two complementary QC measures confirm stable instrument performance and spectral unmixing across the study and distinguish an instrument fault from a reagent fault. Instrument and mAb fluorescence stability was tracked across 38 acquisition dates spanning approximately 32 months (20231016–20260619). Instrument QC monitored the %CV of each of the eight fluorescent peaks (RB-1 to RB-8) of rainbow calibration beads (RB), relative to the first acquisition of each RB lot (a lot-specific baseline). mAb fluorescence QC monitored the fluorescence intensity (FI) of experimental mAb bound to SpectraComp beads across all instrument detectors (the signatures used for spectral unmixing) relative to a fixed reference derived from multiple early dates to encompass all experimental fluorescence. Dissimilarity was quantified as 1 − cosine similarity between each date’s detector profile (FI or %CV) and the reference, capturing shape change independent of magnitude (0 = identical, 1 = orthogonal). **(A)** Dot-plot heatmaps of mAb spectral-signature dissimilarity (left; per fluorochrome) and rainbow-bead %CV dissimilarity (right; per bead peak, RB-1 to RB-8) for each QC date (rows); circle area and color both encode dissimilarity (scales at right). Rows and columns are ordered by hierarchical clustering. **(B, C)** Detector-level detail for the most deviated fluorochrome and bead peak identified by the clustering in **(A)**, on the two flagged dates (Bad date 1, 20240501; Bad date 2, 20250808), each compared with a representative good date (20260619) and the stable reference (20250620): **(B)** per-detector FI for PE and PE-Fire 810 single-stain controls, and **(C)** per-detector %CV of rainbow-bead peak RB-4. Median dissimilarity across the study was 0.0014 (mAb spectra) and 0.023 (bead %CV); the largest single values (0.200 and 0.419, respectively) both occurred on 20240501. Dates were flagged by inspection of the clustered dissimilarity profiles, with no automated numerical threshold applied; numeric labels are shown for dissimilarities ≥ 0.05 (mAb panel) and ≥ 0.25 (RB panel) to highlight the largest changes in each metric.

Both measures were generally highly stable across the study. mAb spectral signatures barely deviated from the reference (**Figures 1A**, left), and rainbow-bead %CV profiles were similarly consistent (**Figures 1A**, right). Against this stable baseline, two acquisition dates stood out and each exemplified one failure mode. The 20240501 date showed the largest deviation in both measures, with markedly elevated mAb spectral dissimilarity for several long-wavelength fluorochromes (e.g. PE-Fire 810, PE-Cy7, and APC-Fire 750) and the highest RB-CV dissimilarities of the study (e.g. RB-4 and RB-5) (**Figures 1A–C**). This concordant pattern suggested an instrument-level fault and coincided with a corrupted instrument file the onboard QC workflow had not flagged. A second date (20250808) showed a smaller mAb spectral change for PE-Fire 810 that occurred with no accompanying rainbow-bead deviation (**Figures 1A–C**), suggesting the fault was the mAb: PE-Fire 810 shifted toward PE-like emission spectrum, characteristic of tandem-dye breakdown (**Figure 1B**).

Both deviations were date-specific rather than progressive drift. Tracking the two measures together therefore both confirmed instrument and mAb spectral signature stability and, through their concordant-versus-dissociated pattern, distinguished an instrument fault from a mAb fault, each pointing to a distinct corrective action (file recovery versus reagent replacement).

### PaCMAP gating identifies leukocyte subsets more reproducibly than traditional 2D biaxial gating

Dimensionality reduction such as PaCMAP (15) can reduce the subjectivity of manual gating by resolving populations as discrete clusters, but its sensitivity to stochastic initialization raises the question of whether those assignments are reproducible across independent experiments (15, 16). To address this, we compared PaCMAP-based (**Supplementary Figures S1B, C**) and traditional 2D biaxial (**Supplementary Figures S1B, D**) gating for longitudinal stability and inter-operator reproducibility, scored against gates drawn in a separately computed UMAP embedding as a reference (**Supplementary Figures S1B, E-G**). It should be noted that UMAP is itself subject to stochastic initialization and is therefore not an absolute biological ground truth. As such, temporal stability and inter-operator agreement were treated as the primary readouts — a common reference still supports these since any bias in it applies equally to both methods — with absolute levels of correct assignment and contamination reported as secondary readouts. Three expert operators independently gated eight major leukocyte populations from CD-CHEX–stabilized human blood (CD-CHEX, whose fixed cellular composition means any operator disagreement reflects gating variability rather than biological change) across up to four timepoints from two donors. Correct assignment was defined as the percentage of cells from each gated population falling within the corresponding UMAP reference gate, and contamination as the percentage falling within any non-corresponding UMAP gate (**Supplementary Figures S1F, G**).

Both methods showed comparable temporal stability of correct assignment, measured as the standard deviation (SD) of assignment percentages across timepoints (PaCMAP, 1.09 vs 2D, 0.78 SD; p = 0.332; **Figures 2A, B, D**). However, PaCMAP gating was substantially more reproducible between operators: the inter-operator intraclass correlation coefficient (ICC; 1 = perfect agreement) for correct assignment was 0.969 for PaCMAP versus 0.742 for 2D (p < 0.001; **Figure 2D**). Both methods were therefore self-consistent over time within an operator, but PaCMAP produced markedly more concordant results between operators.

**Figure 2:**
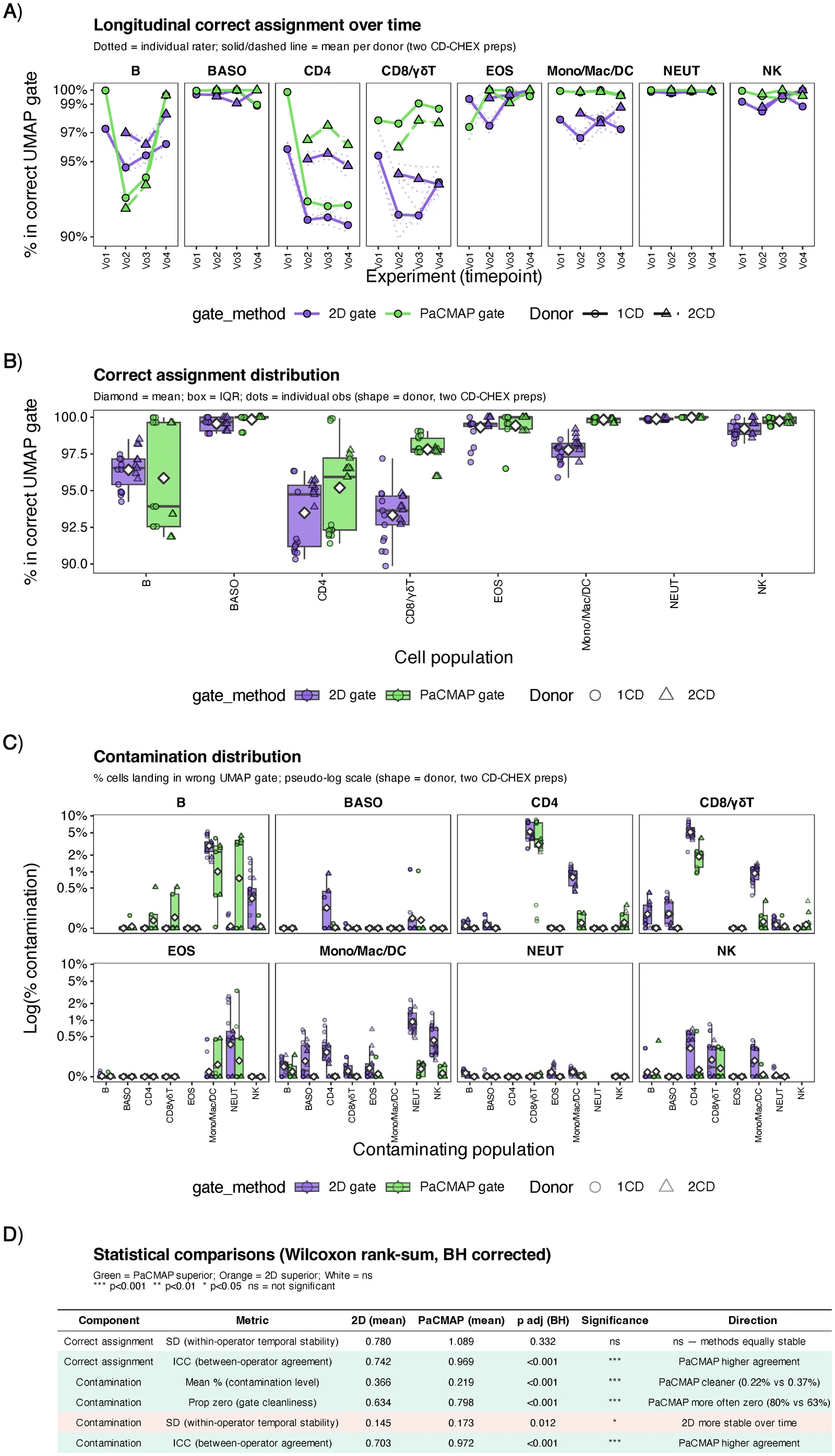
Comparison of PaCMAP-based and traditional 2D biaxial gating consistency for leukocyte subset identification. CD-CHEX–stabilised blood from two donors (different lots; CD1 and CD2) was gated across up to four experimental timepoints by three independent expert operators using either traditional 2D hierarchical biaxial gating (purple) or PaCMAP dimensionality-reduction-based gating (green). Gating purity was assessed against UMAP-defined reference gates drawn by a single expert. **(A)** Longitudinal trajectories of correct-assignment percentages (% cells landing in the corresponding UMAP reference gate) across timepoints (Vo1 to Vo4), faceted by population (columns); the two donors are distinguished within each facet by point shape (circle, triangle) and mean-line style (solid, long-dashed). Each bold line links the per-donor mean at each timepoint (filled points, colored by method); dotted light lines are individual operator trajectories (largely overlapping and difficult to discern for PaCMAP). The y-axis is pseudo-log-scaled to resolve values near 100% (using scales::pseudo_log_trans(sigma = 0.1) in R). **(B)** Boxplots of correct-assignment percentages per population; boxes show the interquartile range (IQR), whiskers extend to 1.5 × IQR, white diamonds mark the group mean, and dots are individual operator × timepoint observations, colored by method and shaped by donor. **(C)** Boxplots of contamination percentages (% cells from a gated population landing in a non-corresponding UMAP reference gate) faceted by UMAP gated population, on a pseudo-log axis to accommodate the many zero values; boxes and dots as in **(B)**, with donor shown by point shape. **(D)** Summary of Wilcoxon rank-sum comparisons (Benjamini–Hochberg-adjusted) between methods across six metrics: correct-assignment temporal stability (SD), correct-assignment inter-rater ICC, mean contamination, proportion of zero-contamination events, contamination temporal stability (SD) and contamination inter-rater ICC; columns give each method’s group mean, the adjusted p-value and the direction; cell color indicates the significant direction (green, PaCMAP superior; orange, 2D superior; white, p ≥ 0.05). ICC (intraclass correlation coefficient) was computed with a two-way model, absolute agreement, single unit (irr::icc); groups with undefined between-operator variance were excluded as degenerate (fewer than two timepoints or three raters, or near-zero overall variance [SD < 0.01]; groups with identical values across raters were treated as perfect agreement), leaving n = 15 (PaCMAP) and n = 16 (2D) for correct assignment. Temporal-stability SD, mean contamination and the zero-contamination proportion were computed per operator and then compared between methods, isolating within-operator consistency; ICC uses the three operators as raters and therefore quantifies between-operator agreement.

PaCMAP gating also produced cleaner gates, with a mean off-target (contamination) rate of 0.22% versus 0.37% for 2D gating (p < 0.001), and more frequently produced gates entirely free of off-target cells (PaCMAP, 79.8% vs 2D, 63.4% of zero contamination; p < 0.001) (**Figures 2C, D**). Contamination patterns were again far more reproducible between operators with PaCMAP compared to 2D gating (ICC 0.972 vs 0.703 respectively; p < 0.001; **Figure 2D**). The single metric on which 2D gating held a marginal advantage was the temporal stability of contamination by each operator (0.15% vs 0.17%; p = 0.012; **Figure 2D**).

Of the six comparisons, five favored PaCMAP or showed equivalence, the sole 2D advantage being marginally more temporally stable contamination within an operator (**Figure 2D**). Dimensionality reduction did not, however, cleanly delineate finer subpopulations, evident in the lack of resolvable white space within the eight major population clusters (**Supplementary Figures S1C, H**), for which manual biaxial gating remained necessary. We therefore adopted a hybrid strategy, PaCMAP-based gating of the major lineages followed by 2D partitioning of their subpopulations (**Supplementary Figure S1H**), for longitudinal donor sample analysis.

### Minimizing pre-analytical and inter-assay variation in longitudinal immunophenotyping

#### Leukocyte composition tolerates chemical fixation and antibody pre-storage but is altered by processing delay and cryopreservation

Longitudinal studies compare samples collected months apart, which may introduce confounding batch-to-batch variance. This can be resolved by banking preserved cells for later batched analysis or mitigated by processing cells fresh each time while stabilizing the other sources of between-run variation; instrument performance and spectral unmixing (above) and the antibody cocktail, prepared and stored in advance rather than made fresh each run. Both routes depend on the manipulation not itself distorting leukocyte composition. To define which compromises are acceptable, we processed whole blood from healthy donors under four pre-analytical conditions and quantified the effect on leukocyte subset composition using multivariate analysis of variance (PERMANOVA) (**Figures 3A–D**).

**Figure 3:**
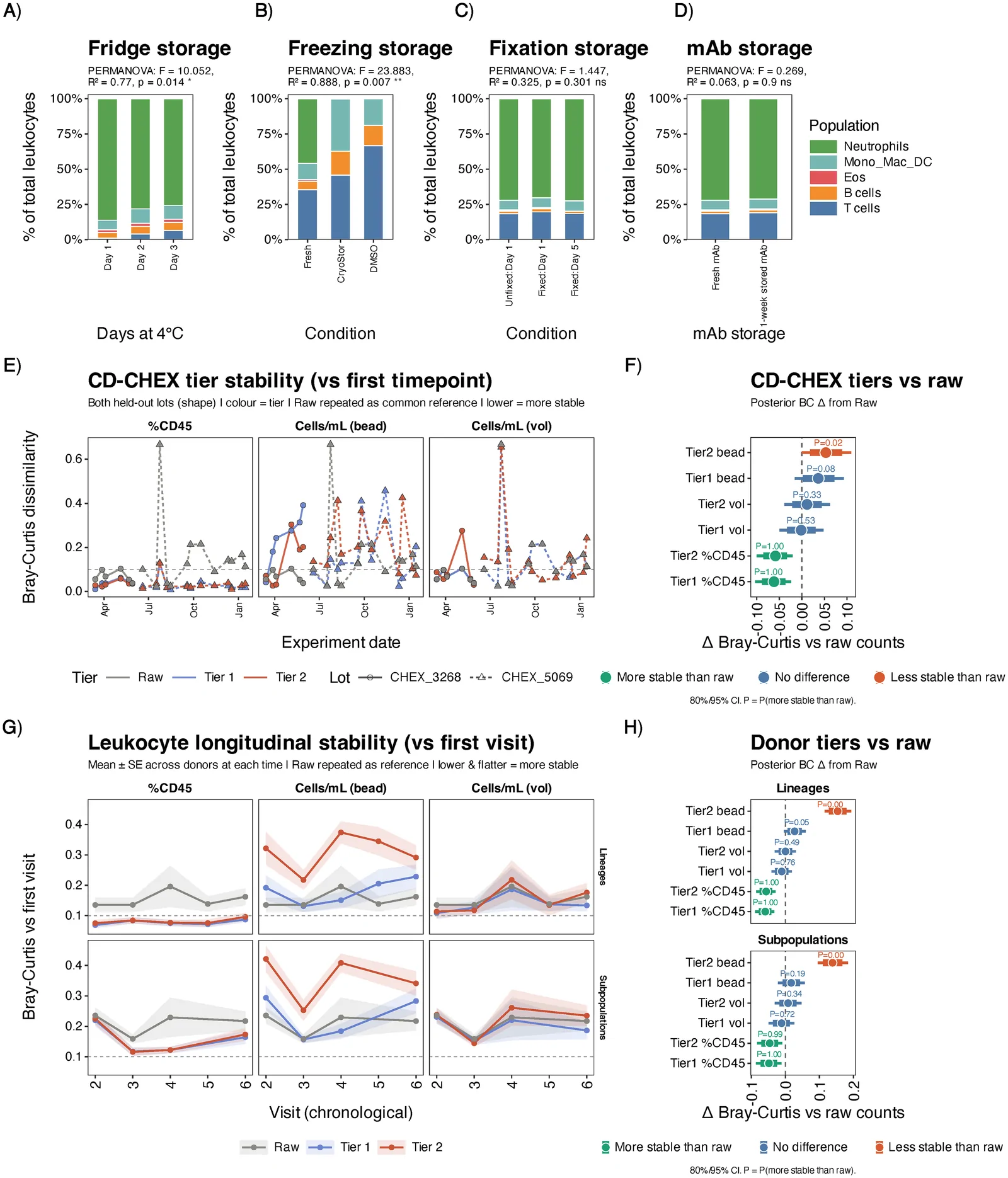
Pre-analytical handling effects on leukocyte composition and two-tier normalization. Whole blood from healthy donors was processed under varying pre-analytical conditions (n = 3 replicates per condition) and immunophenotyped by flow cytometry; populations are expressed as a percentage of total leukocytes. **(A–D)** Mean leukocyte subset composition (stacked bars: neutrophils, monocytes/macrophages/dendritic cells, eosinophils, B cells, T cells) per condition, with the five-population Bray–Curtis PERMANOVA result above each panel: **(A)** refrigerated storage at 4°C for 1, 2 or 3 days (Day 1 fresh reference; samples were pooled then split from donor 1, 4, and 6); **(B)** cryopreservation in CryoStor CS10 or DMSO for 15 days versus fresh (samples were split from donor 4); **(C)** chemical fixation analyzed at Day 1 or Day 5 versus unfixed Day 1 (samples were split from donor 4); **(D)** one-week-stored versus freshly prepared antibody cocktail (samples were split from donor 4). Companion data of numerical mean values in **Supplementary Data S4**. **(E–H)** Two sequential leukocyte count normalization tiers were compared against raw counts for longitudinal stability. Tier 1 rescaled within-sample (to CD45^+^ % [%CD45], or to cells/mL by counting beads [bead] or volume [vol]); Tier 2 added a CD-CHEX-anchored cross-batch correction to remove run-to-run drift (schemes detailed in **Supplementary Figure S2**): **(E)** Lineage populations of CD-CHEX, including eight major lineage populations (as described in **Supplementary Figure S1C**), were assessed for count stability over time (y-axis, Bray–Curtis dissimilarity to the first timepoint per tier, x-axis, acquisition month; lower and flatter = more stable). Each line is one normalization scheme (color) for one held-out CD-CHEX lot (line style and point shape distinguish the two lots, CHEX 3268 and CHEX 5069), with raw counts (raw) shown in every facet as a common reference; facets are the three Tier families (%CD45, bead, vol). The dashed line marks a dissimilarity of 0.1 for reference. **(F)** Posterior contrasts of CD-CHEX lineage count stability for each scheme versus raw counts (model below). **(G)** Longitudinal stability of lineage and subpopulation counts from fresh blood of 10 donors (populations as in **Supplementary Figure S1H**). X-axis, visit in chronological order; y-axis, mean Bray–Curtis dissimilarity to the first visit across donors, with the shaded ribbon the standard error across donors; each line is one scheme (color), raw counts repeated in each facet as reference; facets are level (lineages, subpopulations) × Tier family. **(H)** Corresponding posterior contrasts versus raw counts. In **(F, H)**, Bray–Curtis dissimilarity to the reference timepoint was modelled by Beta regression (brms) with normalization scheme as a fixed effect and a random intercept per population group (CD-CHEX lot in F; donor in H). Points are the posterior median difference from raw counts (ΔBray–Curtis) and bars the 80% (thick) or 95% (thin) credible interval (CI); negative values indicate greater stability than raw. A scheme was classed as more stable (green) or less stable (orange) than raw when its 95% CI excluded zero, and equivalent (gray) when it included zero. P denotes the posterior probability that a scheme has lower temporal dissimilarity than raw (the proportion of posterior draws with ΔBray–Curtis < 0), such that P = 1.00 indicates every draw favored greater stability. All models converged well (Rˆ ≤ 1.01, no divergent transitions); model convergence and fit diagnostics are given in **Supplementary Data S2**.

Delaying processing by storing whole blood at 4°C for two or three days significantly altered subset composition (p = 0.014; **Figure 3A**), driven by progressive neutrophil loss (86.2% fresh to 78.2% and 75.7% at Days 2 and 3) with a reciprocal lymphoid rise, consistent with preferential granulocyte loss during refrigerated storage (33) (**Supplementary Data S4**). Cryopreservation also had a significant effect (**Figure 3B**): both CryoStor and DMSO-based methods caused near-complete loss of neutrophils and eosinophils with marked relative increase in T cells and monocytes/macrophages/dendritic cells (p = 0.007), consistent with selective granulocyte death during freeze–thaw (9). In contrast, chemical fixation preserved major-lineage composition (fixed samples at Day 1 or Day 5 were indistinguishable from unfixed Day 1 blood (p = 0.301; **Figure 3C**)), as did antibody cocktails stored for one week at 4°C (p = 0.900; **Figure 3D**). However, fixation has been reported to distort finer subpopulations, including CD4, regulatory T, CD8, monocyte and basophil subsets (11), precisely the resolution our 44-marker panel is designed to capture. We therefore defaulted to fresh-cell analysis, made practical by the week-long stability of pre-prepared mAb cocktails, with fixation retained only as a contingency.

#### %CD45 normalization improves longitudinal stability whereas counting-bead normalization degrades it

Having fixed the pre-analytical route, we asked whether residual inter-assay variability — that is, variation between separate runs and batches of the same assay — could be further reduced by count normalization. We assessed a panel of schemes across two sequential Tiers. Tier 1 involved a within-sample rescaling in one of three ways: i) rescaling to total CD45^+^ leukocytes (%CD45), ii) rescaling to acquired counting beads (bead), or iii) rescaling to total acquired volume (vol). Tier 2 added a cross-batch correction anchoring lineage and subpopulation counts to CD-CHEX stabilized leukocytes acquired alongside each sample. We evaluated each scheme’s effect on longitudinal stability in two independent datasets, held-out CD-CHEX lots (**Figure 3E**; independent stability controls) and repeated fresh sampling from the same donors (**Figure 3G**), measuring cell quantity drift as the Bray–Curtis dissimilarity of each timepoint from the first, for both lineages and finer subpopulations. We also assessed if the schemes were credibly different from raw counts within a Bayesian Beta model, expressing each result as the posterior difference in dissimilarity from raw counts with its 95% credible interval (CI), and as the posterior probability of being more stable than raw counts (P) (**Figures 3F, H**).

In both datasets, %CD45 normalization was credibly more stable than raw counts across both tiers (lineage data had P = 1.00 **Figures 3F, H**-top panel; and subpopulation data had P ≥ 0.999 **Figure 3H**-bottom panel). In contrast, volumetric normalization (both Tiers) and Tier 1 bead normalization were indistinguishable from raw counts, their 95% CIs spanning zero (**Figures 3F, H**). Furthermore, Tier 2 bead normalization was credibly less stable than raw counts, its 95% CI lying entirely above zero (**Figures 3F, H**). %CD45 normalization therefore improved the temporal stability of leukocyte quantification, whereas counting-bead normalization added, rather than removed, longitudinal variability.

### Random-forest re-identification confirms %CD45 normalization and subpopulation features best preserve individual donor signatures

The ultimate purpose of the pipeline is to preserve true leukocyte signatures faithfully enough across time so that they can serve as predictive biomarkers of disease states such as cancer. We therefore used machine-learning re-identification of individual donors as a functional test of that fidelity: a scheme that better preserves a person’s signature should permit more accurate donor prediction. Ten donors were sampled across six visits over approximately one year, and random-forest classifiers were trained to predict donor identity for a held-out visit (leave-one-visit-out cross-validation; chance accuracy 0.10), separately for each normalization scheme and for three feature sets (leukocyte main lineages, subpopulations, or both combined; **Figure 4**).

**Figure 4:**
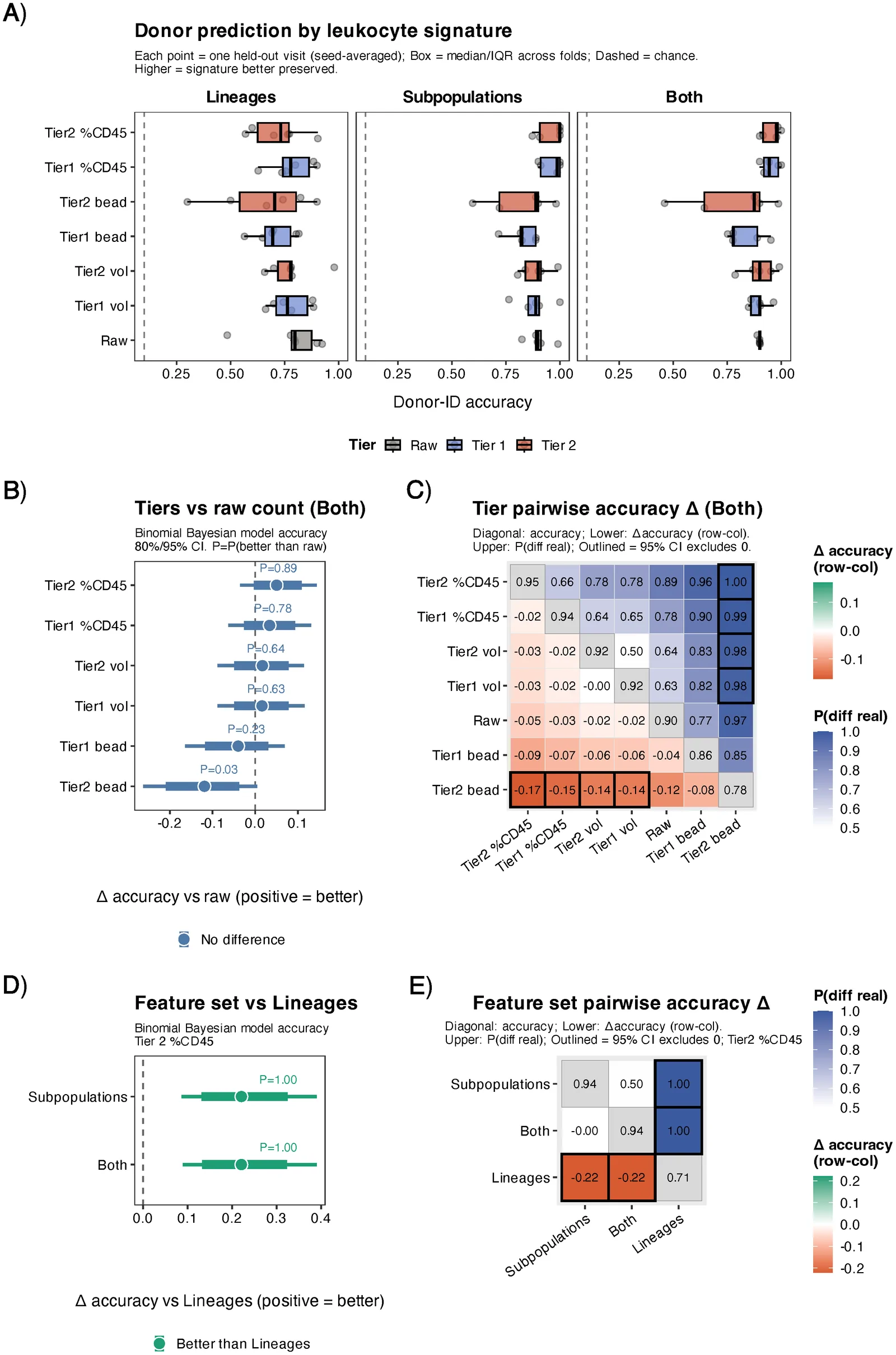
Normalization method and feature set shape random-forest re-identification of individual donors from leukocyte signatures. Random-forest classifiers (ranger) were trained to predict donor identity for a held-out visit (leave-one-visit-out; 10 donors, 6 visits over ~12 months, 25 seeds per condition; chance = 0.10, dashed line). **(A)** Donor-identification accuracy for each normalization method (Raw; Tier 1/Tier 2 × %CD45/bead/volume), shown separately for three feature sets (leukocyte main lineages, subpopulations, both combined); boxes show median/IQR across folds of seed-averaged accuracy, points are individual held-out-visit predictions (averaged across 25 seeds). **(B)** Posterior estimates (Bayesian binomial model, brms; random intercept per held-out visit) of the accuracy difference of each method versus raw counts (combined feature set); points are posterior medians, bars the 80% (thick) or 95% (thin) credible intervals (CI), and P is the posterior probability that the method is *more accurate than raw*; “No difference” denotes a 95% CI crossing zero. **(C)** Pairwise accuracy differences among normalization methods (combined feature set): lower triangle, median accuracy difference (row − column); upper triangle, P(difference real) — the posterior probability the difference is nonzero in its observed direction; outlined cells have a 95% CI excluding zero. **(D)** Accuracy difference of the subpopulation and combined feature sets versus lineages alone (Tier 2%CD45); points, bars and P as in **(B)**. **(E)** Pairwise feature-set differences (Tier 2%CD45) displayed as in **(C)**. All models converged well (Rˆ ≤ 1.01, no divergent transitions); convergence and fit diagnostics are given in **Supplementary Data S3**. Companion numerical values are in **Supplementary Tables S2** and **S3**.

Re-identification accuracy far exceeded chance for every scheme but varied systematically with both feature set and normalization scheme (**Figure 4A**). The %CD45 schemes gave consistently the highest accuracy, particularly when subpopulations were included in the feature set (**Figure 4A**, middle and right panels). %CD45 accuracy using subpopulations alone was 0.960 (Tier 1) and 0.956 (Tier 2), and the combined feature set behaved similarly (Tier 2 accuracy 0.955; Tier 1 accuracy 0.950; **Figure 4A**, **Supplementary Table S2**). Cross-validated accuracy ranks the schemes but does not indicate if their differences are larger than the uncertainty in the estimates themselves. To assess whether the normalization schemes were credibly different in accuracy we therefore used a Bayesian binomial model to capture sampling noise while accounting for variation at the level of the held-out visit. Bayesian binomial contrasts showed that, although %CD45 normalization tended to outperform raw counts, this difference was not credible (its 95% CI crossed zero); indeed, no normalization scheme was credibly different from raw counts (**Figure 4B**). Credible differences did, however, emerge between schemes: pairwise comparison using the combined feature set revealed Tier 2 bead normalization to be the worst performer (combined-feature accuracy 0.773), credibly worse than the %CD45 and volumetric schemes (P = 0.977–0.997; **Figure 4C**, **Supplementary Table S3**). Of all pairwise comparisons against this poorest benchmark, Tier 2%CD45 showed the largest accuracy improvement and the highest probability of a real difference (**Figure 4C**, **Supplementary Table S3**). Bead-based normalization therefore appears to add instability to the leukocyte signature rather than removing it, while Tier 2%CD45 ranked highest across the pairwise comparisons.

A random forest can separate donors using non-linear combinations of leukocyte population quantities, so it can succeed even where the measured values themselves drift. We therefore repeated the re-identification with a deterministic nearest-centroid classifier, which assigns each held-out visit to the donor whose remaining visits it most closely resembles (**Supplementary Figure S3**). Because that assignment depends on the measured values staying closer to the donor’s own center than to anyone else’s, it tests longitudinal stability directly. Accuracy using this approach tracked that of the random forest across every normalization scheme and feature set (**Supplementary Figure S3A**), with no credible difference between the two classifiers for any scheme (**Supplementary Figure S3B**). The deterministic classifier also resolved a difference the random forest did not: both counting-bead schemes were credibly worse than raw counts (**Supplementary Figure S3C**), whereas no scheme was credibly separated from raw under the random forest (**Figure 4B**). At Tier 1%CD45 with the combined feature set, 46 of 48 held-out donor-visits were assigned to the correct donor (**Supplementary Figure S3D**). The individual leukocyte signature is therefore recoverable by direct similarity to a donor’s own remaining visits, and does not depend on a non-linear learner.

The choice of feature set also mattered: donor signatures built from subpopulations carried more individual-discriminating information than broad lineages alone. For the best-performing scheme (Tier 2%CD45), adding subpopulation features, alone or combined with lineages, credibly improved classification accuracy by +0.22 over lineage-only models (P = 1.00; **Figures 4D, E**, **Supplementary Table S4**).

Taken together, the optimized pipeline with percentage-of-CD45 normalization using subpopulation-level features preserved individual leukocyte signatures well enough to re-identify donors from a single held-out visit with high accuracy. Given the small donor panel, we interpret this re-identification not as a standalone diagnostic claim but as functional evidence that the pipeline preserves true biological signal.

### Blood leukocyte signatures are dominated by stable, individually distinct inter-donor differences

The high donor re-identification accuracy above implies that individual leukocyte signatures are both distinct between people and stable within a person over time, particularly when appropriate variability controls and high-resolution subpopulation data are used. To test this directly, we analyzed the immune signatures of 10 donors sampled every two months across a 12-month period, using Tier 2%CD45 normalization and the combined leukocyte feature set. We visualized the distinct signatures by hierarchical clustering of mean leukocyte composition (heatmap), decomposed the compositional variance into between- and within-donor contributions (Bray–Curtis PERMANOVA), and compared inter- with intra-donor leukocyte composition variation using multivariate dispersion and principal-coordinates analysis (PCoA) (**Figure 5**).

**Figure 5:**
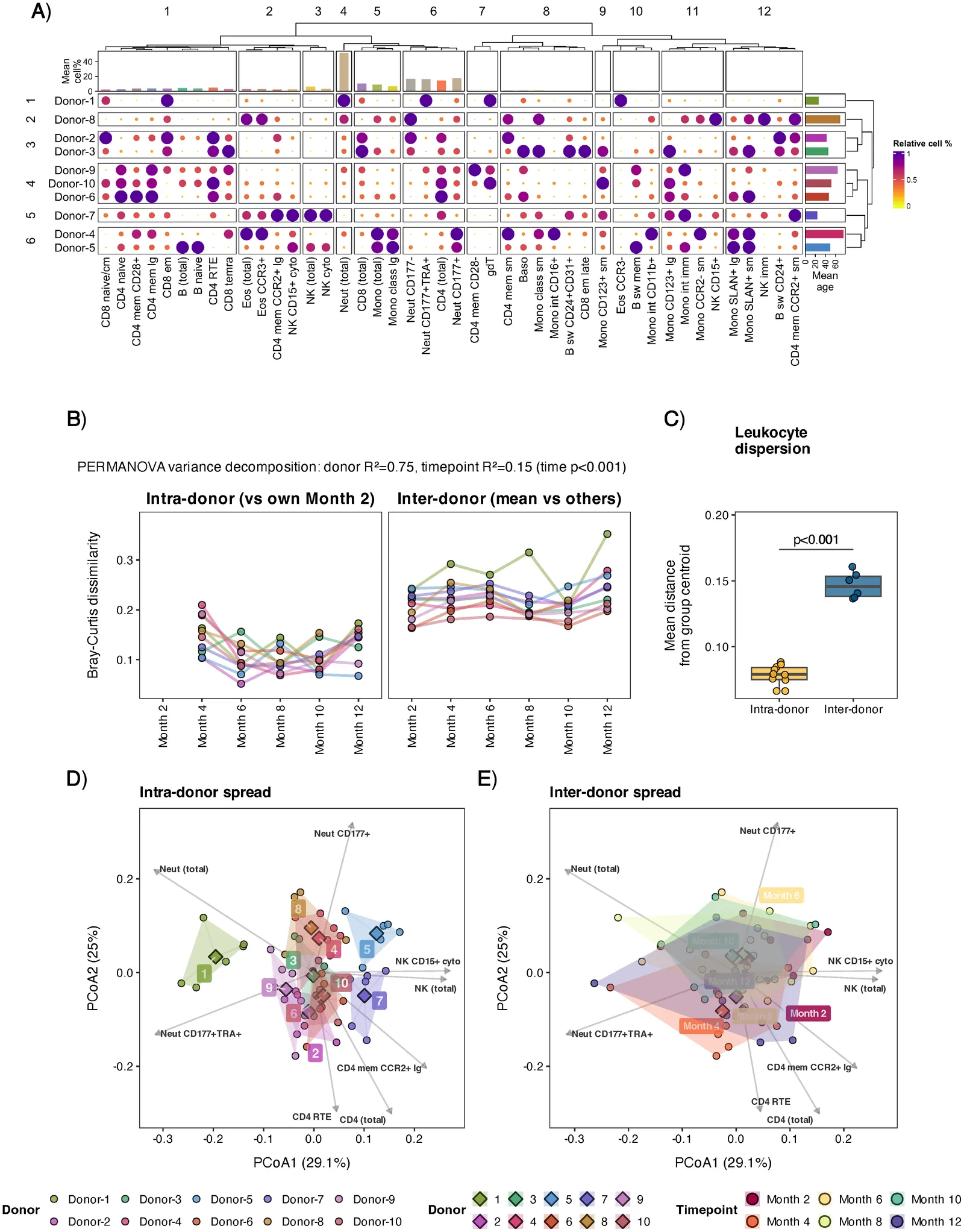
Blood leukocyte signatures are dominated by stable, individually distinct inter-donor differences. Longitudinal leukocyte composition for 10 donors across six visits (58 samples; two donations missing — donor 8 at the final visit and donor 10 at the fourth), using Tier 2%CD45 normalisation and the combined leukocyte feature set (population names defined in **Supplementary Data S1**). **(A)** Heatmap of relative leukocyte population abundance (populations in columns, donors in rows); circle area scaled to per-column 0–1 relative abundance, with population and donor dendrograms (Bray–Curtis, Ward.D2) and donor-cell-mean-abundance and mean-age annotations. **(B)** Bray–Curtis dissimilarity across the leukocyte signature space per donation timepoint: (left) intra-donor, each donor relative to their own first visit; (right) inter-donor: for each donor, the mean of its Bray–Curtis dissimilarities to every other donor at the same visit; colored points are individual donors (see donor color key). The subtitle reports a PERMANOVA variance decomposition of leukocyte composition (composition ~ timepoint + donor; Bray–Curtis; marginal terms), where R² is the proportion of total compositional variance explained by each term. Multivariate dispersions were homogeneous among donors and timepoints (betadisper permutation: donor F = 0.343, p = 0.965; timepoint F = 0.366, p = 0.883), confirming the **(B)** differences reflect composition rather than unequal spread. **(C)** Mean distance to the group centroid in the full Bray–Curtis leukocyte signature space, for intra- versus inter-donor groupings; inter-donor dispersion (0.147) exceeded intra-donor dispersion (0.078) (group-level one-sided permutation test, p < 0.001). **(D, E)** The same contrast as **(C)** visualized in the two most variance-explaining principal coordinates: PCoA (Bray–Curtis) of all samples colored by donor, with convex hulls showing intra-donor spread — each donor’s own visits **(D)** — and inter-donor spread — donors at each timepoint **(E)**; axes give the percentage of variance explained by each coordinate. Grey arrows are leukocyte populations fitted to the ordination (envfit, 999 permutations); the eight populations most strongly associated with the PCoA axes (p ≤ 0.05, ranked by strength of association) are shown, arrow direction indicating increasing abundance. The same vectors appear in **(D, E)**, which share one PCoA.

Hierarchical clustering revealed markedly heterogeneous global leukocyte compositions across donors and many subpopulations (**Figure 5A**). Multivariate analysis across the total leukocyte signature space revealed within-donor dissimilarity from the initial time of sampling was less than the between-donor dissimilarity (**Figure 5B** left vs right panel) and PERMANOVA analysis confirmed that donor identity was the overwhelmingly dominant source of variance, explaining 75% of leukocyte-compositional variance versus 15% attributable to sampling time (PERMANOVA; **Figure 5B**). Although composition did shift detectably within each donor over time (timepoint term p < 0.001), donor identity accounted for roughly five times as much variance, so who a sample came from mattered far more than when it was taken.

Consistent with this, permutation tests showed that between-donor dispersion across the leukocyte signature space exceeded within-donor dispersion 1.9-fold (p < 0.001; **Figure 5C**). Visualizing this in the first two coordinates of a PCoA highlighted that individual donors occupied distinct, largely non-overlapping regions of the signature space across all their visits (**Figure 5D**, hulls), whereas the same samples grouped by timepoint overlapped extensively (**Figure 5E**, hulls), confirming that each donor’s repeat visits cluster together while donors remain separated. Leukocyte populations fitted to this space (arrows indicating the direction of increasing abundance) showed that donor separation was driven by a mix of lineages: neutrophil (including CD177^+^ and CD177^+^TRA^+^ subsets), NK, and CD4 T-cell (CD4 recent thymic emigrant and CCR2^+^ memory) populations (envfit p ≤ 0.05; **Figures 5D, E**). Individuality was therefore distributed across the immune compartment rather than confined to a single lineage, spanning both innate (neutrophil, NK) and adaptive (CD4 T-cell) populations.

Together these data show that an individual’s blood leukocyte composition constitutes a temporally stable, individually distinct signature — an immunological fingerprint — within which donor identity dwarfs the variation introduced by sampling at different times. This directly explains the donor re-identification result (**Figure 4**, **Supplementary Figure S3**). Therefore, an instrument shown to be stable (**Figure 1**), gated reproducibly (**Figure 2**), with composition preserved through appropriate handling and normalization (**Figure 3**), recovers biological leukocyte signatures dominated by stable inter-individual differences (**Figures 4**, **5**). This suggests the pipeline is suitable for longitudinal immune monitoring.

## Discussion

We set out to build and validate an optimized pipeline for longitudinal blood leukocyte immunophenotyping, and to test whether a person’s leukocyte signature is stable and distinct enough between individuals to be biologically meaningful. We found: 1) Instrument performance and spectral unmixing were stable across more than two years of analysis, using a dual-measure QC metric capable of flagging and identifying sources of machine or reagent failures (**Figure 1**); 2) PaCMAP-based gating of major lineages was substantially more reproducible between operators than manual 2D gating, and produced cleaner gates, while remaining temporally stable (**Figure 2**); 3) Leukocyte composition tolerated chemical fixation and one-week antibody pre-storage, but was altered by delayed processing and profoundly restructured by cryopreservation (**Figure 3**); 4) Percentage-of-CD45 normalization best preserved longitudinal compositional stability (**Figure 3**), while counting-bead normalization was credibly less stable (**Figure 3**) and gave the poorest donor re-identification among schemes (**Figure 4**); and, finally, 5) the recovered signatures were dominated by stable, individually distinct inter-donor differences, sufficient to predict donor identity both from a trained classifier and from simple distance to a donor’s own signature center (**Figure 4**, **Supplementary Figure S3**, **Figure 5**).

The need for rigorous quality control in high-parameter and spectral cytometry is well recognized, with standardization frameworks emphasizing instrument calibration and longitudinal monitoring (5, 6, 13). Our dual-measure approach adds to these by monitoring not only instrument stability, through rainbow beads, but also reagent integrity, through single-stain spectral signatures, allowing anomalies to be localized to either the instrument or a specific probe: a distinction conventional bead-only QC cannot make. Complementing these acquisition-level controls, we ran CD-CHEX stabilized human blood alongside every sample batch as a biological invariant; because its cellular composition is fixed, it provides a running check on assay stability across the study and a common reference for cross-batch normalization. Similarly, the subjectivity of manual gating is a long-standing concern (7), and dimensionality-reduction methods have been proposed to mitigate it; however, their sensitivity to stochastic initialization raises doubts about reproducibility across experiments (16). We show that PaCMAP-based gating is substantially more reproducible between operators than manual 2D gating, while confirming that it does not resolve finer subpopulations, motivating our hybrid strategy. Our blood sampling preparation results align with prior reports that refrigerated storage and, more severely, cryopreservation selectively deplete granulocytes (8, 9, 33), and that chemical fixation preserves major lineages but distorts finer subpopulations (11); we add that pre-prepared antibody cocktails remain stable for a week, making fresh-cell processing practical across a longitudinal study. Finally, whereas count-normalization strategies are known to disagree (12), we provide a direct longitudinal comparison showing that reference-population (%CD45) normalization best preserves signal, whereas counting-bead normalization actively degrades it. While this suggests leukocyte composition expressed as a percentage of total CD45 is the preferred quantification for longitudinal sampling, one caveat is that a disease state producing a uniform shift in overall leukocyte abundance would be undetectable under such a readout, since relative composition would remain the same. To quantify absolute leukocyte changes we would favor the volumetric normalized measures, which were no less stable than raw counts, unlike the counting-bead normalization schemes, which added rather than removed longitudinal variability. Tier 2 volumetric counts are easily collected in parallel with %CD45 values and carry the CD-CHEX cross-batch correction onto a common scale, making them an appropriate absolute readout to report alongside %CD45 composition where absolute abundance is informative.

The central biological finding is that an individual’s blood leukocyte composition, that is, subset frequencies expressed as a proportion of CD45^+^ leukocytes, forms a temporally stable, distinct signature. This is consistent with evidence that immune composition varies widely between people but is comparatively stable within a person over time (3, 19, 34). Evidence suggests blood cell immune composition is shaped by both heritable and environmental factors: genome-wide studies of ~170,000 people have resolved thousands of largely cell-type-specific loci for white-cell traits, yet age, sex, body mass, smoking and alcohol account for a large share of the remaining variation and even widen the variance of these traits through gene–environment interactions (35, 36). The premise of our study is that layered on top of these already substantial contributors of biological variance are avoidable technical sources of variation. Instrument drift, gating inconsistency, pre-analytical handling, longitudinal assay variation and count normalization are all potential noise generators, each of which must be minimized before an individual signature can be resolved. Our pipeline aimed to address and control these systematically, and resulted in stable, individually distinct signatures.

This finding also frames a pathway for using blood-based immune signatures as biomarkers for disease states such as cancer: a routine, optimized high-parameter assay that maximizes technical reproducibility is the necessary basis for detecting disease-associated change. Despite this, what was also clear, particularly using the donor re-identification analysis and PERMANOVA variance analysis is that between-individual variation is predictably distinct and large, and may overwhelm disease-associated changes if population-based referencing is used (37). Therefore, disease signatures are likely to be most detectable as deviation from a personal or group-matched baseline. Since establishing a personal baseline requires longitudinal history rarely available at diagnosis, individuals grouped on the major determinants of immune composition, such as age, sex and key exposures, may be a promising alternative analogous to controlled preclinical work we have established for cancer (2, 4). Establishing such baselines in humans will require larger longitudinal cohorts, but depends first on the reproducible, low-noise measurement this pipeline provides.

Indeed, our study has clear limitations in this regard, as the donor panel used was small (ten donors with complete longitudinal sampling), and larger cohorts will be needed to resolve the trends and to establish whether the individual fingerprint is stable over years rather than months. Furthermore, while we tried to resolve one of the most practical limitations of using flow cytometry – subjective gating – with a dimensionality reduction-based strategy, such approaches have limited scope in delineating finer subpopulations that we found vital to signature predictive value. This issue will hopefully be resolved with future development of innovative unsupervised clustering algorithms (38, 39). Finally, the longitudinal, normalization and re-identification analysis were all performed at a single site using one spectral instrument, one mAb panel with unmixing reference sets of a single type throughout. Transfer across sites, instrument platforms or panel designs was not assessed and would require separate validation.

These results provide a practical, validated basis for longitudinal blood leukocyte immunophenotyping within a uniform spectral flow cytometry framework. Having tested each component, we now apply the full pipeline as standard for our human blood work: rainbow-bead and mAb-spectra quality control, fresh-cell labelling with pre-stored antibody cocktails, PaCMAP-based lineage gating, and Tier 2%CD45 normalization. With avoidable technical noise minimized, the biology that dominates is the individual’s own signature, and perturbations around that signature may, in turn, signal disease. With this foundation, blood leukocyte signatures could support accessible, repeatable biomarkers of immune state in cancer and other diseases (2, 4).

## Data Availability

The datasets presented in this study can be found in online repositories. The names of the repository/repositories and accession number(s) can be found below: All data and analysis code underlying the findings of this study are publicly available in a self-contained research compendium deposited in the Zenodo repository: 10.5281/zenodo.21366352. The repository contains the consolidated source data and the R scripts required to reproduce figures and statistical results in the article.

## Supplementary material

Supplementary Figure 1 Gating strategies.

Supplementary Figure 2 Two-tier normalisation schematic.

Supplementary Figure 3 Nearest-centroid doner re-identification

Supplementary Table 1 Monoclonal antibody panel.

Supplementary Table 2 Donor re-identification performance by normalisation method.

Supplementary Table 3 Credible pairwise differences in donor re-identification accuracy.

Supplementary Table 4 Feature-set comparison versus lineages alone.

Supplementary Data Sheet 1 Leukocyte subset name key.

Supplementary Data Sheet 2 Two-tier normalisation Bayesian model diagnostics.

Supplementary Data Sheet 3 Donor classification performance Bayesian model diagnostics.

Supplementary Data Sheet 4 Sample-handling composition means (**Figures 3A–D**).
