# Supplementary material for: An optimized spectral flow cytometry pipeline for longitudinal blood leukocyte immunophenotyping reveals stable, individually distinct immune signatures
Source: Front Immunol. 2026 Oct 5;17:1959895. doi: 10.3389/fimmu.2026.1959895 (PMC13677906; doi:10.3389/fimmu.2026.1959895)
Supplement: Supplementary file 1 [file Image1.pdf]

S1 Fig. Population and bead gating strategies.

(A) Rainbow Calibration Particles (rainbow beads [RB]) were gated initially using scatter parameters as a cleaning step, followed by individual peak gates (RB1-RB8) using the V7_A channel.

RBC-depleted human blood cells or CD-CHEX–stabilised human cells were labelled with a 44-marker fluorescent mAb panel, analysed by flow cytometry, and gated using CytoExploreR. (B) Standard cleaning gates (non-debris and bead-exclusion gates, live CD45+ gates [the live gate was not applied to CD-CHEX], single-cell gates); UMAP and PaCMAP dimensionality reduction was performed on the final single, live CD45+ cells using the cyto_map() CytoExploreR function. (C) PaCMAP-based gating used rough lineage gates (top panels) to label PaCMAP clusters (lower left panel), allowing the eight major populations (CD4+ T cells, CD8+/γδ T cells, NK cells, B cells, neutrophils, monocytes/macrophages/dendritic cells, eosinophils, basophils) to be gated directly in PaCMAP space (lower right panel). (D) 2D gating used conventional hierarchical biaxial strategies on the same lineage markers for the same eight populations. (E) The independent reference was built by a single operator applying the same lineage gates (from the 2D lineage overlays; D) directly in UMAP space. (F, G) Each gating method was then scored against that UMAP reference: for (F) PaCMAP-gated and (G) 2D-gated populations, correct assignment was the percentage of cells from each gated population falling within the corresponding UMAP reference gate, and contamination the percentage falling within any non-corresponding UMAP gate. (H) Total adopted gating scheme combining refinements for standard cleaning, rough lineage gates, and PaCMAP-based main population gates combined with manual 1D and 2D gates for subpopulation gating.

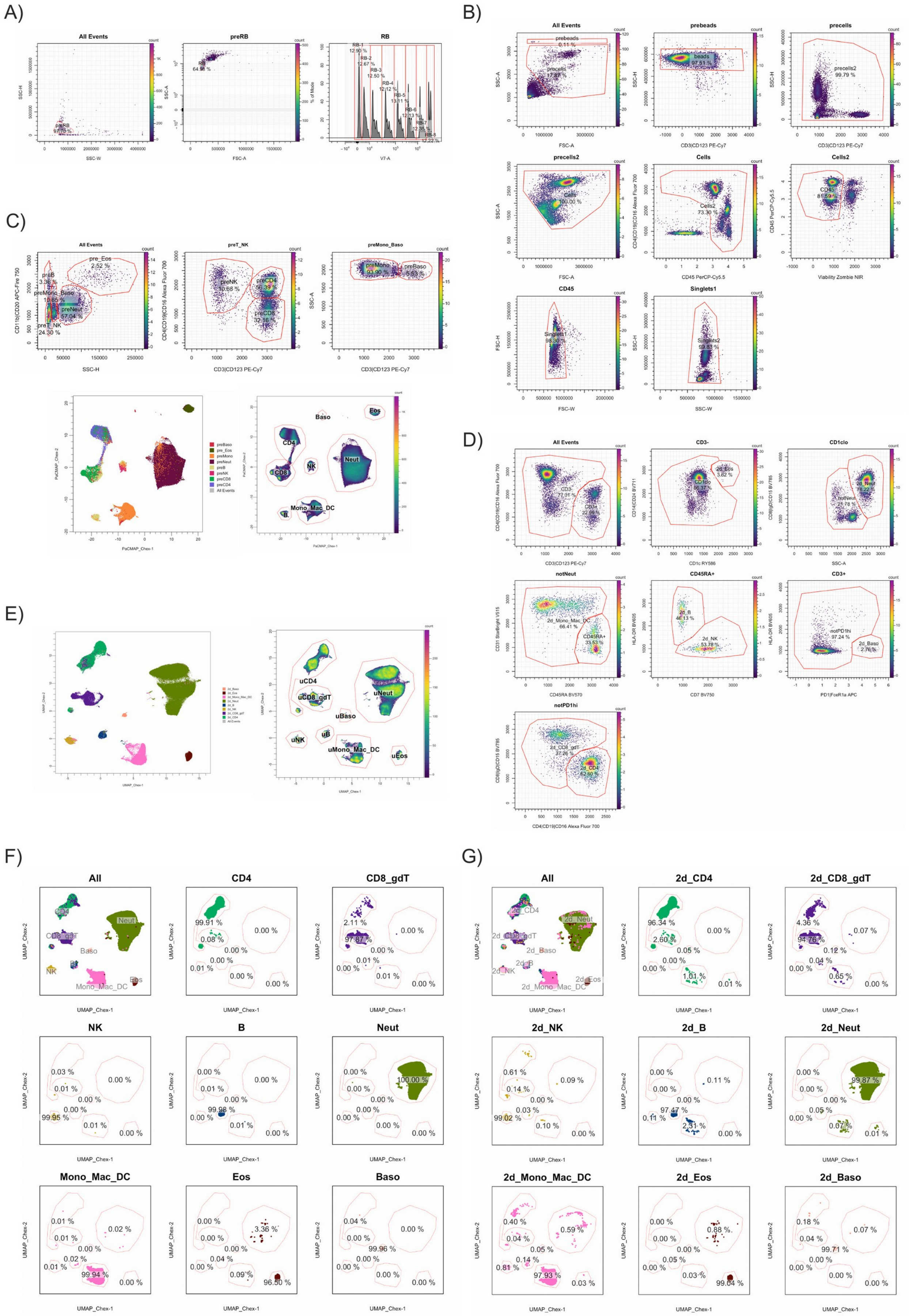
A)
All Events
preRB
RB
B)
All Events
prebeads
precells
precells2
Cells
Cells2
CD45
Singlets1
C)
All Events
preT_NK
preMono_Baso
D)
All Events
CD3-
CD1clo
notNeut
CD45RA+
CD3+
notPD1hi
E)
F)
All
CD4
CD8_gdT
NK
B
Neut
Mono_Mac_DC
Eos
Baso
G)
All
2d_CD4
2d_CD8_gdT
2d_NK
2d_B
2d_Neut
2d_Mono_Mac_DC
2d_Eos
2d_Baso

H)

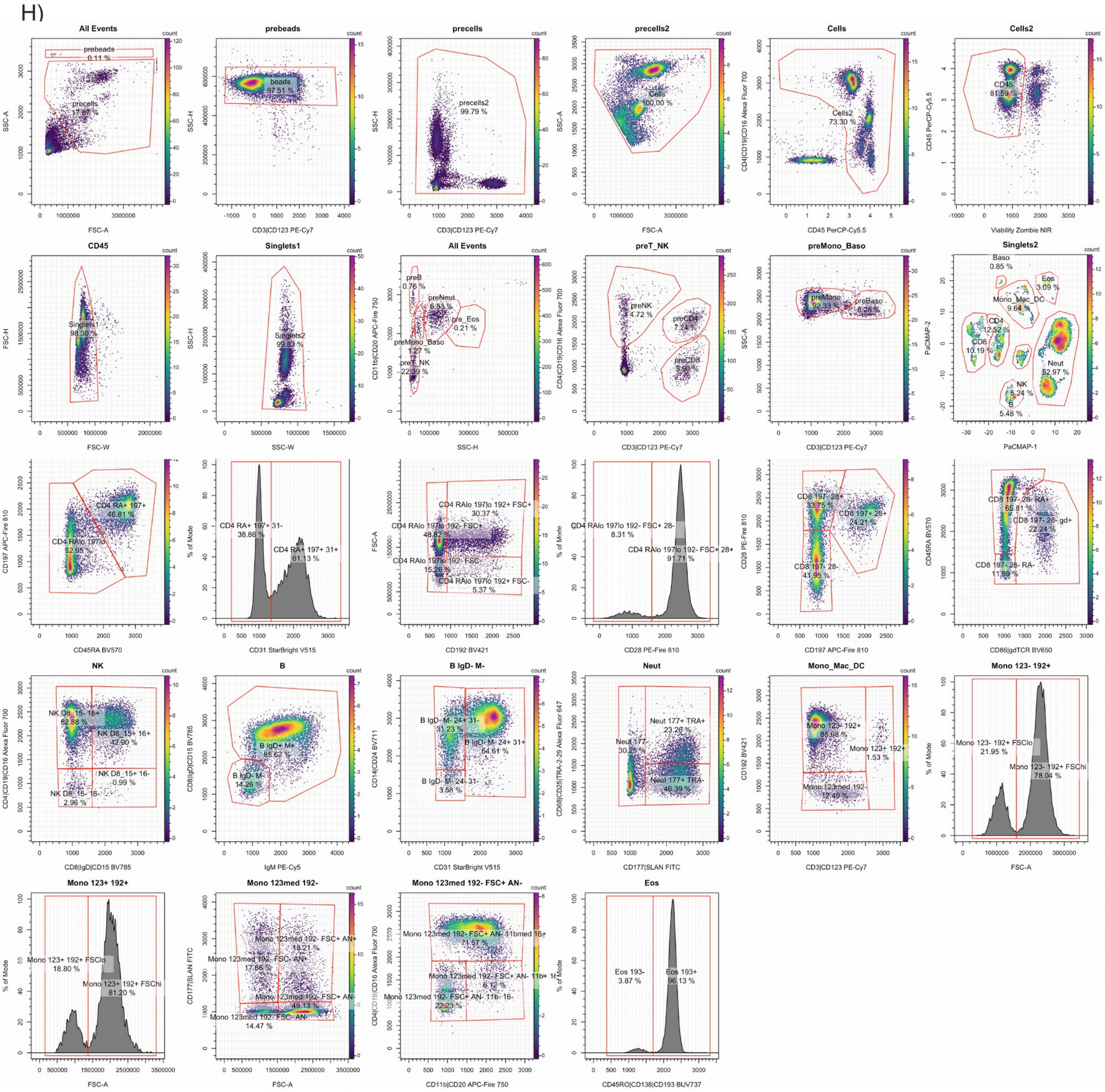
All Events
prebeads
precells
precells2
Cells
Cells2
CD45
Singlets1
All Events
preT_NK
preMono_Baso
Singlets2
NK
B
B IgD- M-
Neut
Mono_Mac_DC
Mono 123- 192+
Mono 123+ 192+
Mono 123med 192-
Mono 123med 192- FSC+ AN-
Eos
