# Supplementary material for: An optimized spectral flow cytometry pipeline for longitudinal blood leukocyte immunophenotyping reveals stable, individually distinct immune signatures
Source: Front Immunol. 2026 Oct 5;17:1959895. doi: 10.3389/fimmu.2026.1959895 (PMC13677906; doi:10.3389/fimmu.2026.1959895)
Supplement: Supplementary file 2 [file Image2.pdf]

Two-tier CD-CHEX longitudinal normalisation

Per-population pipeline: raw counts to CD-CHEX-anchored normalised counts

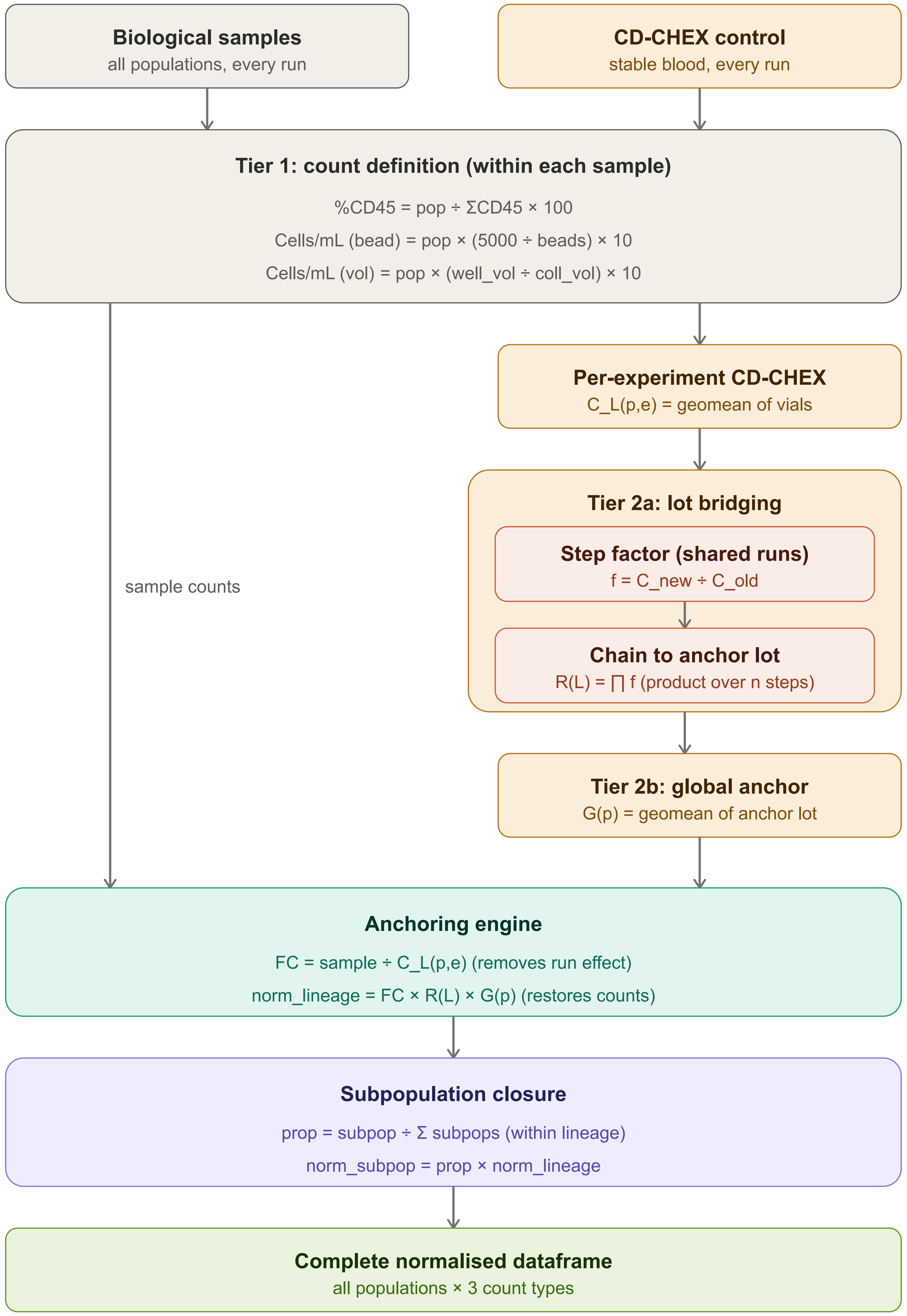

Symbols & abbreviations

CD-CHEX
Stabilised whole-blood control (Streck CD-Chex Plus); invariant, run with every experiment

pop
Raw event count of a population in a sample

ΣCD45
Sum of the 8 main lineages = total CD45+ cells

beads
Counting-bead events; 5000 = beads added per well; ×10 rescales per-100 µL to per mL

well_vol / coll_vol
Acquisition well and blood collection volumes

p, e, L
Population, experiment (run), CD-CHEX lot

C_L(p,e)
Geometric-mean CD-CHEX for lot L, population p, in experiment e (over replicate vials)

f
Per-step bridge factor: ratio of two lots on the runs they shared (C_new ÷ C_old)

∏
Product operator: multiply the listed terms together (here, the step factors f along a chain)

R(L) = ∏ f
Cumulative re-anchor factor: product of the step factors f along the chain back to the anchor lot

G(p)
Global anchor: geometric mean of the anchor lot's values for population p over its runs

FC
Fold change of sample vs same-day CD-CHEX

geomean
Geometric mean (used at every averaging step)

Colour key

- Sample data flowing through
- CD-CHEX correction being built
- Lot-bridging sub-steps
- Anchoring engine
- Subpopulation step
- Final output
