# Supplementary material for: An optimized spectral flow cytometry pipeline for longitudinal blood leukocyte immunophenotyping reveals stable, individually distinct immune signatures
Source: Front Immunol. 2026 Oct 5;17:1959895. doi: 10.3389/fimmu.2026.1959895 (PMC13677906; doi:10.3389/fimmu.2026.1959895)

S3 Fig. Nearest-centroid assignment reproduces the random-forest donor re-identification result.

A deterministic nearest-centroid classifier was applied under the same leave-one-visit-out scheme as Fig 4 (10 donors, 6 visits over ~12 months; chance = 0.10, dashed line). Each feature is one leukocyte population under the scheme being tested. For each held-out visit, features were standardised as z-scores using means and standard deviations taken across the training samples only, pooling donors and visits; a centroid was computed for every donor by averaging the z-values for each population across that donor's training visits, giving one value per population; and the held-out sample was assigned to the nearest of the ten donor centroids by Euclidean distance across all populations. The classifier has no random seed, hyperparameters or tuning step. Both classifiers were scored on identical folds, so their comparison in (B) isolates the decision rule. Panels A and C follow the format of Fig 4A and Fig 4B so that the two classifiers can be compared directly. (**A**) Nearest-centroid accuracy for each normalisation method (raw counts, and Tier 1 and Tier 2 forms of the %CD45, counting-bead and volumetric schemes), shown separately for three feature sets (leukocyte main lineages, subpopulations, both combined); each point is one held-out visit and boxes show median/IQR across folds. (**B**) Posterior difference in accuracy between the two classifiers, nearest centroid minus random forest, estimated from a single model fitted to both classifiers on identical folds (combined feature set); points are posterior medians and bars the 80% (thick) and 95% (thin) credible intervals. A credible interval spanning zero indicates the two classifiers are indistinguishable on these folds, which was the case for every normalisation scheme. (**C**) Posterior accuracy difference of each normalisation method versus raw counts (combined feature set), from a Bayesian binomial model of the fold-level counts with the held-out visit as a random intercept; points and bars as in (B), and P is the posterior probability that the method is more accurate than raw. (**D**) Assignment matrix at Tier 1 %CD45 with the combined feature set: rows are the true donor, columns the assigned donor, and cell labels the number of held-out visits placed in that cell. Both Bayesian models converged well (maximum R-hat 1.001, no divergent transitions).

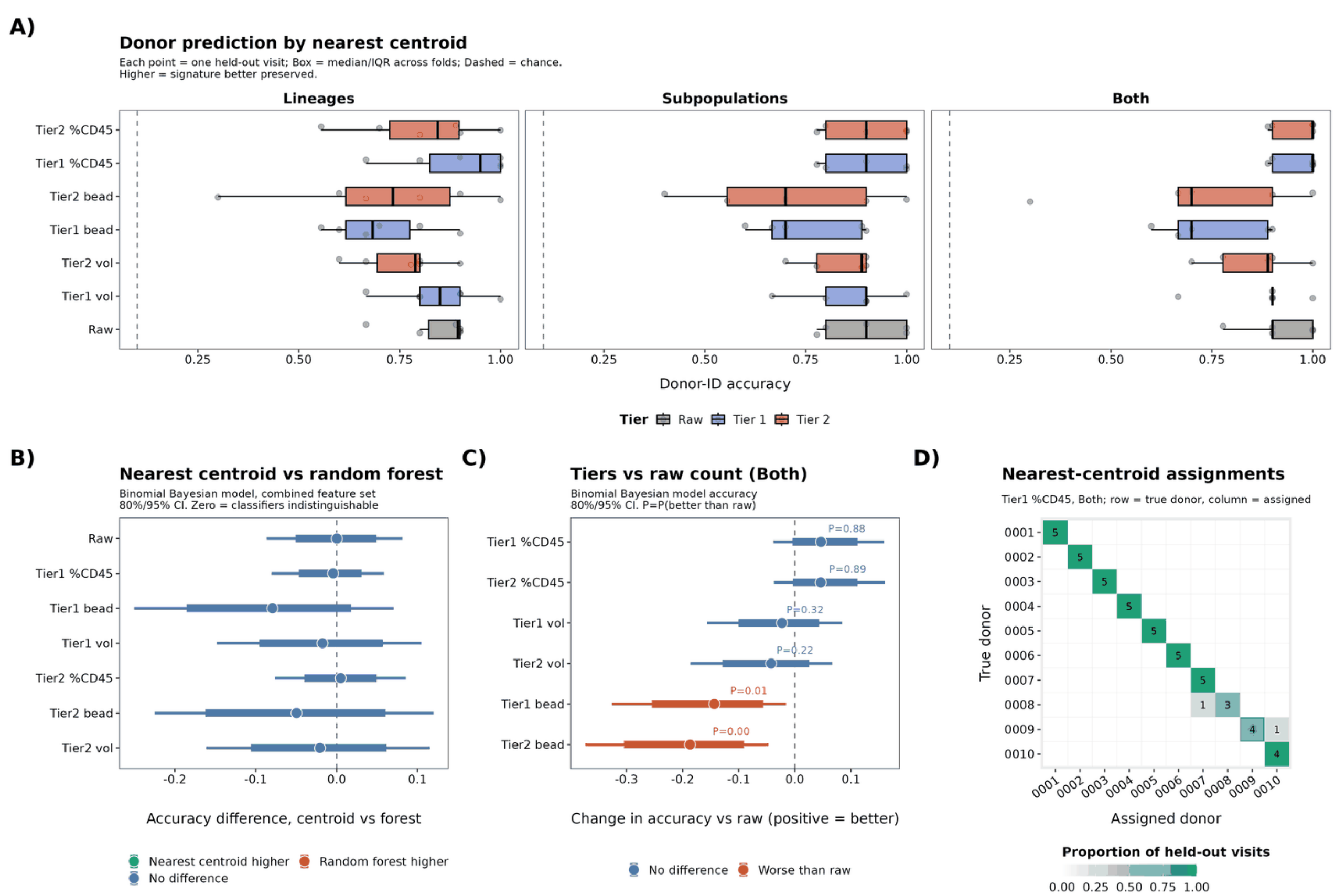

Supplement: Supplementary file 3 [file Image3.pdf]
