# Supplementary material for: An optimized spectral flow cytometry pipeline for longitudinal blood leukocyte immunophenotyping reveals stable, individually distinct immune signatures
Source: Front Immunol. 2026 Oct 5;17:1959895. doi: 10.3389/fimmu.2026.1959895 (PMC13677906; doi:10.3389/fimmu.2026.1959895)
Supplement: Supplementary file 4 [file Table1.docx]

## S1 Table: Monoclonal antibody panel used in the study

| **Antigen** | **Clone** | **Fluorochrome** | **Isotype** | **Cat. #** | **Source** | ***Final conc (1/X)*** |
| --- | --- | --- | --- | --- | --- | --- |
| CCR2 (CD192) | K036C2 | BV421 | Mouse IgG2a, k | 357210 | Biolegend | 200 |
| CXCR1 (CD181) | 5A12 | BV480 | Mouse IgG2b, k | 746798 | BD | 200 |
| CD31 | WM59 | StarBright Violet 515 | Mouse IgG1 | MCA1738SBV515 | BioRad | 100 |
| CD62L | DREG-56 | BV510 | Mouse IgG1, κ | 304844 | Biolegend | 300 |
| CD11c | S-HCL-3 | BV510 | Mouse IgG2b, κ | 371514 | Biolegend | 200 |
| CD45RA | HI100 | BV570 | Mouse IgG2b, k | 304132 | Biolegend | 400 |
| HLA-DR | L243 | BV605 | Mouse IgG2a, κ | 307640 | Biolegend | 80 |
| CD86 | IT2.2 | BV650 | Mouse IgG2b, κ | 305428 | Biolegend | 200 |
| gdTCR | 11F2 | BV650 | Mouse IgG1 | 745359 | BD | 200 |
| CD14 | M5E2 | BV711 | Mouse IgG2a, κ | 301838 | Biolegend | 400 |
| CD24 | ML5 | BV711 | Mouse IgG2a, κ | 311136 | Biolegend | 200 |
| CD7 | 4H9 | BV750 | Mouse BALB/c IgG1, k | 753766 | BD | 200 |
| CD8 | SK1 | BV785 | Mouse IgG1, κ | 344740 | Biolegend | 600 |
| IgD | IA6-2 | BV785 | Mouse IgG2a, κ | 348242 | Biolegend | 200 |
| CD15 | W6D3 | BV785 | Mouse IgG1, κ | 323044 | Biolegend | 200 |
| CD177 | MEM166 | FITC | Mouse IgG1, k | 315804 | Biolegend | 200 |
| SLAN (M-DC8) | DD-1 | FITC | Mouse IgM, k | 130-117-371 | Miltenyi | 400 |
| CD49d | L25 | RB545 | Mouse BALB/c IgG2b, k | 756362 | BD | 200 |
| CD1c | F10/21A3 | RY586 | Mouse IgG1, k | 753662 | BD | 100 |
| PDL1 | 29E.2A3 | PE-Daz594 | Mouse IgG2b, κ | 329732 | Biolegend | 200 |
| CD127 | AO19D5 | PE-Daz594 | Mouse IgG1, κ | 351336 | Biolegend | 40 |
| KLRG1 | 2F1/KLRG1 | PE-Fire 640 | Syrian Hamster IgG | 138440 | Biolegend | 50 |
| IgM | G20-127 | PE-Cy5 | Mouse IgG1, k | 551079 | BD | 100 |
| CD45 | HI30 | PerCP-Cy5.5 | Mouse IgG1, κ | 982312 | Biolegend | 100 |
| CD27 | LG.7F9 | PerCP-eFluor 710 | Armenian Hamster IgG | 46-0271-82 | Thermo | 400 |
| CD56 | TULY56 | cFluor BYG750 | Mouse IgG1, k | SKU R7-20284 | Cytek | 50 |
| CD3 | SK7 | PE-Cy7 | Mouse IgG1, κ | 981010 | Biolegend | 1000 |
| CD123 | 6H6 | PE-Cy7 | Mouse IgG1, κ | 983702 | Biolegend | 200 |
| CD28 | CD28.2 | PE-Fire 810 | Mouse IgG1, k | 302971 | Biolegend | 400 |
| PD1 | A17188A | APC | Mouse IgG2b, κ | 379208 | Biolegend | 100 |
| FceR1a | AER-37 | APC | Mouse IgG2b, κ | 334612 | Biolegend | 80 |
| CD25 | BC96 | AF647 | Mouse IgG1, κ | 302642 | Biolegend | 80 |
| CD68 | Y1/82A | AF647 | Mouse BALB/c IgG2b, κ | 562111 | BD | 50 |
| TRA-2-49 | TRA-2-49/6E | AF647 | Mouse IgG1, k | 358710 | Biolegend | 50 |
| CD117 (c-Kit) | 104D2 | Spark NIR 685 | Mouse IgG1, k | 313250 | Biolegend | 200 |
| CD4 | RPA-T4 | AF700 | Mouse IgG1, κ | 300526 | Biolegend | 1000 |
| CD19 | SJ25C1 | AF700 | Mouse IgG1, κ | 363034 | Biolegend | 40 |
| CD16 | 3G8 | AF700 | Mouse IgG1, κ | 302026 | Biolegend | 200 |
| CD45RO | UCHL1 | BUV737 | Mouse BALB/c IgG2a, κ | 748368 | BD | 200 |
| CD138 | MI15 | BUV737 | Mouse BALB/c IgG1, κ | 612834 | BD | 200 |
| CD193 | 5E8 | BV737 | Mouse C57BL/6 IgG2b, κ | 612853 | BD | 200 |
| CD11b | M1/70 | APC-F750 | r-IgG2b, k | 101262 | Biolegend | 80 |
| CD20 | 2H7 | APC-F750 | Mouse IgG2b, κ | 302358 | Biolegend | 400 |
| CCR7 (CD197) | G043H7 | APC-Fire 810 | Mouse IgG2a, k | 353264 | Biolegend | 200 |

## S2 Table: Donor-re-identification performance by normalisation method

| **feature_set** | **metric** | **measure** | **mean** | **ci_low** | **ci_high** | **n_evaluations** |
| --- | --- | --- | --- | --- | --- | --- |
| Lineages | Tier1 %CD45 | Accuracy | 0.784 | 0.600 | 0.900 | 150 |
| Lineages | Raw | Accuracy | 0.780 | 0.444 | 1.000 | 150 |
| Lineages | Tier2 vol | Accuracy | 0.780 | 0.667 | 1.000 | 150 |
| Lineages | Tier1 vol | Accuracy | 0.775 | 0.667 | 0.900 | 150 |
| Lineages | Tier2 %CD45 | Accuracy | 0.718 | 0.556 | 0.900 | 150 |
| Lineages | Tier1 bead | Accuracy | 0.704 | 0.556 | 0.900 | 150 |
| Lineages | Tier2 bead | Accuracy | 0.655 | 0.300 | 0.900 | 150 |
| Subpopulations | Tier1 %CD45 | Accuracy | 0.960 | 0.889 | 1.000 | 125 |
| Subpopulations | Tier2 %CD45 | Accuracy | 0.956 | 0.800 | 1.000 | 125 |
| Subpopulations | Raw | Accuracy | 0.902 | 0.700 | 1.000 | 125 |
| Subpopulations | Tier2 vol | Accuracy | 0.890 | 0.778 | 1.000 | 125 |
| Subpopulations | Tier1 vol | Accuracy | 0.882 | 0.700 | 1.000 | 125 |
| Subpopulations | Tier1 bead | Accuracy | 0.827 | 0.700 | 0.900 | 125 |
| Subpopulations | Tier2 bead | Accuracy | 0.817 | 0.600 | 1.000 | 125 |
| Both | Tier2 %CD45 | Accuracy | 0.955 | 0.889 | 1.000 | 125 |
| Both | Tier1 %CD45 | Accuracy | 0.950 | 0.889 | 1.000 | 125 |
| Both | Tier2 vol | Accuracy | 0.900 | 0.778 | 1.000 | 125 |
| Both | Raw | Accuracy | 0.898 | 0.800 | 1.000 | 125 |
| Both | Tier1 vol | Accuracy | 0.895 | 0.778 | 1.000 | 125 |
| Both | Tier1 bead | Accuracy | 0.829 | 0.667 | 1.000 | 125 |
| Both | Tier2 bead | Accuracy | 0.773 | 0.400 | 1.000 | 125 |
| Lineages | Tier2 vol | Top-3 accuracy | 0.979 | 0.900 | 1.000 | 150 |
| Lineages | Raw | Top-3 accuracy | 0.965 | 0.889 | 1.000 | 150 |
| Lineages | Tier1 %CD45 | Top-3 accuracy | 0.948 | 0.889 | 1.000 | 150 |
| Lineages | Tier1 vol | Top-3 accuracy | 0.948 | 0.889 | 1.000 | 150 |
| Lineages | Tier2 %CD45 | Top-3 accuracy | 0.931 | 0.800 | 1.000 | 150 |
| Lineages | Tier1 bead | Top-3 accuracy | 0.929 | 0.889 | 1.000 | 150 |
| Lineages | Tier2 bead | Top-3 accuracy | 0.843 | 0.400 | 1.000 | 150 |
| Subpopulations | Tier1 %CD45 | Top-3 accuracy | 1.000 | 1.000 | 1.000 | 125 |
| Subpopulations | Tier2 %CD45 | Top-3 accuracy | 1.000 | 1.000 | 1.000 | 125 |
| Subpopulations | Tier1 bead | Top-3 accuracy | 0.977 | 0.889 | 1.000 | 125 |
| Subpopulations | Raw | Top-3 accuracy | 0.959 | 0.889 | 1.000 | 125 |
| Subpopulations | Tier2 bead | Top-3 accuracy | 0.948 | 0.800 | 1.000 | 125 |
| Subpopulations | Tier2 vol | Top-3 accuracy | 0.938 | 0.889 | 1.000 | 125 |
| Subpopulations | Tier1 vol | Top-3 accuracy | 0.937 | 0.889 | 1.000 | 125 |
| Both | Tier2 %CD45 | Top-3 accuracy | 0.998 | 1.000 | 1.000 | 125 |
| Both | Tier1 %CD45 | Top-3 accuracy | 0.995 | 0.900 | 1.000 | 125 |
| Both | Raw | Top-3 accuracy | 0.974 | 0.889 | 1.000 | 125 |
| Both | Tier1 bead | Top-3 accuracy | 0.963 | 0.889 | 1.000 | 125 |
| Both | Tier2 vol | Top-3 accuracy | 0.939 | 0.889 | 1.000 | 125 |
| Both | Tier1 vol | Top-3 accuracy | 0.934 | 0.889 | 1.000 | 125 |
| Both | Tier2 bead | Top-3 accuracy | 0.934 | 0.700 | 1.000 | 125 |
| Lineages | Raw | Macro-F1 | 0.733 | 0.407 | 1.000 | 150 |
| Lineages | Tier1 %CD45 | Macro-F1 | 0.730 | 0.483 | 0.867 | 150 |
| Lineages | Tier2 vol | Macro-F1 | 0.721 | 0.593 | 1.000 | 150 |
| Lineages | Tier1 vol | Macro-F1 | 0.709 | 0.593 | 0.867 | 150 |
| Lineages | Tier2 %CD45 | Macro-F1 | 0.661 | 0.517 | 0.867 | 150 |
| Lineages | Tier1 bead | Macro-F1 | 0.626 | 0.481 | 0.867 | 150 |
| Lineages | Tier2 bead | Macro-F1 | 0.590 | 0.173 | 0.867 | 150 |
| Subpopulations | Tier1 %CD45 | Macro-F1 | 0.946 | 0.852 | 1.000 | 125 |
| Subpopulations | Tier2 %CD45 | Macro-F1 | 0.941 | 0.733 | 1.000 | 125 |
| Subpopulations | Raw | Macro-F1 | 0.870 | 0.600 | 1.000 | 125 |
| Subpopulations | Tier2 vol | Macro-F1 | 0.853 | 0.704 | 1.000 | 125 |
| Subpopulations | Tier1 vol | Macro-F1 | 0.844 | 0.600 | 1.000 | 125 |
| Subpopulations | Tier1 bead | Macro-F1 | 0.776 | 0.617 | 0.867 | 125 |
| Subpopulations | Tier2 bead | Macro-F1 | 0.763 | 0.483 | 1.000 | 125 |
| Both | Tier2 %CD45 | Macro-F1 | 0.941 | 0.852 | 1.000 | 125 |
| Both | Tier1 %CD45 | Macro-F1 | 0.934 | 0.852 | 1.000 | 125 |
| Both | Tier2 vol | Macro-F1 | 0.866 | 0.704 | 1.000 | 125 |
| Both | Raw | Macro-F1 | 0.864 | 0.733 | 1.000 | 125 |
| Both | Tier1 vol | Macro-F1 | 0.861 | 0.704 | 1.000 | 125 |
| Both | Tier1 bead | Macro-F1 | 0.778 | 0.574 | 1.000 | 125 |
| Both | Tier2 bead | Macro-F1 | 0.707 | 0.233 | 1.000 | 125 |

Donor-re-identification performance by normalisation method (accuracy, top-3 accuracy and macro-F1; mean and 2.5-97.5 percentile spread per feature set).

## S3 Table: Credible pairwise differences in donor-re-identification accuracy

| **better** | **worse** | **accuracy_gap** | **ci_low** | **ci_high** | **prob_difference_real** |
| --- | --- | --- | --- | --- | --- |
| Tier2 %CD45 | Tier2 bead | 0.170 | 0.049 | 0.316 | 0.997 |
| Tier1 %CD45 | Tier2 bead | 0.153 | 0.026 | 0.302 | 0.991 |
| Tier2 vol | Tier2 bead | 0.135 | 0.002 | 0.282 | 0.977 |
| Tier1 vol | Tier2 bead | 0.135 | 0.003 | 0.285 | 0.977 |

Credible pairwise differences in donor-re-identification accuracy between normalisation methods (combined feature set; only contrasts whose 95% CI excludes zero), with median accuracy gap and P(direction).

## S4 Table: Feature-set comparison versus lineages alone

| **tier** | **feature_set** | **accuracy_vs_lineages** | **ci_low** | **ci_high** | **prob_better_than_lineages** | **verdict** |
| --- | --- | --- | --- | --- | --- | --- |
| Tier1 %CD45 | Subpopulations | 0.147 | 0.031 | 0.300 | 0.992 | Better than Lineages |
| Tier1 %CD45 | Both | 0.129 | 0.005 | 0.281 | 0.980 | Better than Lineages |
| Tier2 %CD45 | Subpopulations | 0.221 | 0.087 | 0.390 | 0.999 | Better than Lineages |
| Tier2 %CD45 | Both | 0.221 | 0.090 | 0.390 | 1.000 | Better than Lineages |

Accuracy of subpopulation and combined feature sets relative to lineages alone, per top normalisation tier, with credible-interval verdict.
